# Evaluating land use and climate change impacts on Ravi river flows using GIS and hydrological modeling approach

**DOI:** 10.1038/s41598-024-73355-2

**Published:** 2024-09-27

**Authors:** Sami Ullah, Usman Ali, Muhammad Rashid, Saif Haider, Ozgur Kisi, Dinesh Kumar Vishwakarma, Ali Raza, Abed Alataway, Ahmed Z. Dewidar, Mohamed A. Mattar

**Affiliations:** 1 Hydraulics and Irrigation Engineering Division, Civil Engineering Department, University of Engineering and Technology, Lahore, 54890 Pakistan; 2 Centre of Excellence in Water Resources Engineering, University of Engineering and Technology, Lahore, 54890 Pakistan; 3 Department of Civil Engineering, Lübeck University of Applied Sciences, 23562 Lübeck, Germany; 4 Department of Civil Engineering, Ilia State University, Tbilisi, 0162 Georgia; 5 Department of Irrigation and Drainage Engineering, Govind Ballabh Pant University of Agriculture and Technology, Pantnagar, Uttarakhand 263145 India; 6 School of Agricultural Engineering, Jiangsu University, Zhenjiang, 212013 People’s Republic of China; 7 Prince Sultan Bin Abdulaziz International Prize for Water Chair, Prince Sultan Institute for Environmental, Water and Desert Research, King Saud University, P.O. Box 2454 Riyadh 11451, Saudi Arabia; 8 Department of Agricultural Engineering, College of Food and Agriculture Sciences, King Saud University, P.O. Box 2454 Riyadh 11451, Saudi Arabia; 9 Agricultural Engineering Research Institute (AEnRI), Agricultural Research Centre, Giza 12618, Egypt

**Keywords:** Land cover, Climate change, River Ravi, Balloki Flows, Water, CMhyd, TerrSet, Hydrological modeling, Climate change, Hydrology

## Abstract

The study investigates the interplay of land use dynamics and climate change on the hydrological regime of the Ravi River using a comprehensive approach integrating Geographic Information System (GIS), remote sensing, and hydrological modeling at the catchment scale. Employing the Soil and Water Assessment Tool (SWAT) model, simulations were conducted to evaluate the hydrological response of the Ravi River to both current conditions and projected future scenarios of land use and climate change. This study differs from previous ones by simulating future land use and climate scenarios, offering a solid framework for understanding their impact on river flow dynamics. Model calibration and validation were performed for distinct periods (1999–2002 and 2003–2005), yielding satisfactory performance indicators (NSE, R^2^, PBIAS = 0.85, 0.83, and 10.01 in calibration and 0.87, 0.89, and 7.2 in validation). Through supervised classification techniques on Landsat imagery and TerrSet modeling, current and future land use maps were generated, revealing a notable increase in built-up areas from 1990 to 2020 and projections indicating further expansion by 31.7% from 2020 to 2100. Climate change projections under different socioeconomic pathways (SSP2 and SSP5) were derived for precipitation and temperature, with statistical downscaling applied using the CMhyd model. Results suggest substantial increases in precipitation (10.9 − 14.9%) and temperature (12.2 − 15.9%) across the SSP scenarios by the end of the century. Two scenarios, considering future climate conditions with current and future land use patterns, were analyzed to understand their combined impact on hydrological responses. In both scenarios, inflows to the Ravi River are projected to rise significantly (19.4 − 28.4%) from 2016 to 2100, indicating a considerable alteration in seasonal flow patterns. Additionally, historical data indicate a concerning trend of annual groundwater depth decline (0.8 m/year) from 1996 to 2020, attributed to land use and climate changes. The findings underscore the urgency for planners and managers to incorporate climate and land cover considerations into their strategies, given the potential implications for water resource management and environmental sustainability.

## Introduction

Two important elements that have a considerable impact on hydrological systems are climate and land use changes, particularly on the flow patterns of rivers^1–4^. Climate change profoundly influences hydrological systems by affecting precipitation, evaporation rates, and snowmelt patterns. These alterations directly impact river flows and their seasonal variability. Changes in precipitation can lead to increased flooding and extended periods of drought, posing severe challenges to water resource management, agricultural productivity, and ecosystem health^5^. Several studies have indicated that climate change has influenced the flow patterns of the Ravi River in recent decades^2,6–10^. Land use change refers to converting natural landscapes for human purposes, such as urbanization, agriculture, and deforestation. Human activities, driven by population growth and economic development, have significantly altered land cover across the globe^11^. These changes disrupt the natural balance of ecosystems and often contribute to increased runoff and erosion. Altering land cover has a profound impact on the hydrological cycle. Forests, wetlands, and grasslands act as natural sponges that store and slowly release water, regulating river flows and reducing the risk of floods^9,12–17^. Conversely, urbanization and deforestation reduce water infiltration into the ground, increasing surface runoff and accelerating erosion. This phenomenon can cause flash floods and adversely affect downstream river flow^18–24^.

The Ravi River, a vital tributary of the Indus River in Pakistan, is crucial in supporting agriculture, providing drinking water, and sustaining ecosystems in the region^25^. However, in recent decades, the Ravi River has experienced fluctuations in flow patterns attributed to the interplay between climate change and land use change^7,9,26^. This general introduction aims to shed light on the complex relationship between these two factors and their consequences by concentrating on the catchment of the Ravi River within Lahore city to assess its response considering current and future land use and climate changes^27–29^.A comprehensive strategy combining mitigation and adaptation methods is required to address the effects of climate change and land use change on Ravi River flows at Balloki. To limit the severity of climate change, mitigation measures should concentrate on reducing greenhouse gas emissions. This requires transitioning to renewable energy sources, enhancing energy efficiency, and implementing afforestation and reforestation projects to sequester carbon dioxide^30,31^. Adaptation strategies should enhance the region’s resilience to changing river flow patterns. Implementing sustainable land use practices, preserving and restoring wetlands, and adopting water-efficient agricultural techniques are essential to mitigating the effects of changing land usage^32–34^.

To tackle the considerable climate change challenges in Pakistan, various hydrological models have been extensively used across various watersheds. These models include the Soil and Water Assessment Tool (SWAT)^35^, the MIKE SHE model^36^, the HEC-HMS model, the UBC watershed model^37^, the HBV model^38^, and the SRM model^39^. Moreover, to assess the impacts of climate change and land use change, the SWAT model is employed^2,40–42^. It is regarded as the most suitable tool for analyzing watershed hydrological systems^43^.

Global climate models (GCMs) are crucial for predicting future climate changes^44^, but their coarse spatial resolution limits their accuracy at the basin scale^4^. To understand climate change impacts on water resources, it is vital to assess hydrological responses at the sub-basin level. The Climate Model Inter-comparison Project (CMIP) provides publicly available results from advanced GCMs, with CMIP6 incorporating updated models and emission scenarios^45^. These advancements improve our understanding of future climate impacts on hydrological systems. GCMs are selected based on their ability to provide key inputs—precipitation, maximum, and minimum temperatures—for hydrological models. These inputs are available for five shared socioeconomic pathways (SSPs): SSP1-1.9, SSP1-2.6, SSP2-4.5, SSP3-7.0, and SSP5-8.5 considering various land-use and greenhouse gas scenarios^46,47^. This comprehensive framework integrates climate change mitigation, adaptation, and sustainable development, informing policy responses to global challenges.

In recent decades, the interplay between land use changes and climate dynamics has become a critical area of research, especially concerning their combined effects on river systems. The River Ravi, a vital water resource in South Asia, exemplifies the challenges posed by these environmental shifts. This study leverages advanced Geographic Information Systems (GIS) and hydrological modeling techniques to analyze how land use transformations and climate variability comprehensively impact the flow patterns of the River Ravi. The current study differs from previous studies in simulating multiple future scenarios of land use changes and climate projections. The study provides a robust framework for understanding potential future conditions and their implications on river flow dynamics.

Climate change is one of the prevailing factors affecting the water quality of rivers worldwide and in Pakistan. Lahore is a megacity in Pakistan that is affected by climate change and urbanization, resulting in groundwater depletion^48,49^. Therefore, there is a need to manage this prevailing problem to counter the future shortage of water resources. Pakistan is affected by significant land use and climate change phenomena, which in turn has affected surface and groundwater resources in the country. This effect is more pronounced in major cities such as Lahore, where the aquifer has been threatened for the past few years due to less recharge and more abstraction. Hence, it is crucial to ascertain the extent of historical land use transformations and climate variations in Lahore, predict their future trajectories, and assess their ramifications on water resources.

Thus, this study quantified the land use and climate change in Lahore across historical and projected scenarios, alongside assessing their influence on the flows of the Ravi River at Balloki. The study achieved the following objectives: (a) Identifying present and future trends in land use and climate change within the study area. (b) Evaluating the hydrology and groundwater depletion rate of the Lahore section of the Ravi River using observational data. (c) Examining the effects of land use and climate change on the flow patterns of the Ravi River.

Section 2 provides an overview of utilized datasets, and employed models and approaches. Section 3 presents the results and major findings of the study. Section 4 provides detailed discussion. Section 5 presents the Theoretical and Practical Repercussions and Sect. 6 presents the conclusion and recommendations drawn based on this study.

## Materials and methods

### Study area

Lahore, Pakistan’s second-largest city after Karachi, is the capital of the Punjab Province and is distinguished by a hot, semiarid climate. Geographically, it is located between 31°15’ and 31°45’ N latitude and 74°01’ and 74°39’ E longitude. It is bordered on the north and west by the Sheikhupura District, on the east by Wagah, and on the south by the Kasur District. The Ravi River runs on the western side of the district of Lahore. The Ravi River, one of the five major rivers in the Punjab region, flows through the northern part of Lahore, providing a crucial water source for the city’s inhabitants, agriculture, and industries. The city’s landscape is predominantly urbanized and characterized by a mix of residential, commercial, and industrial areas, green spaces and historical landmarks. Lahore areas are surrounded by agricultural lands contributing to the region’s food production and economy.

**Fig. 1:**
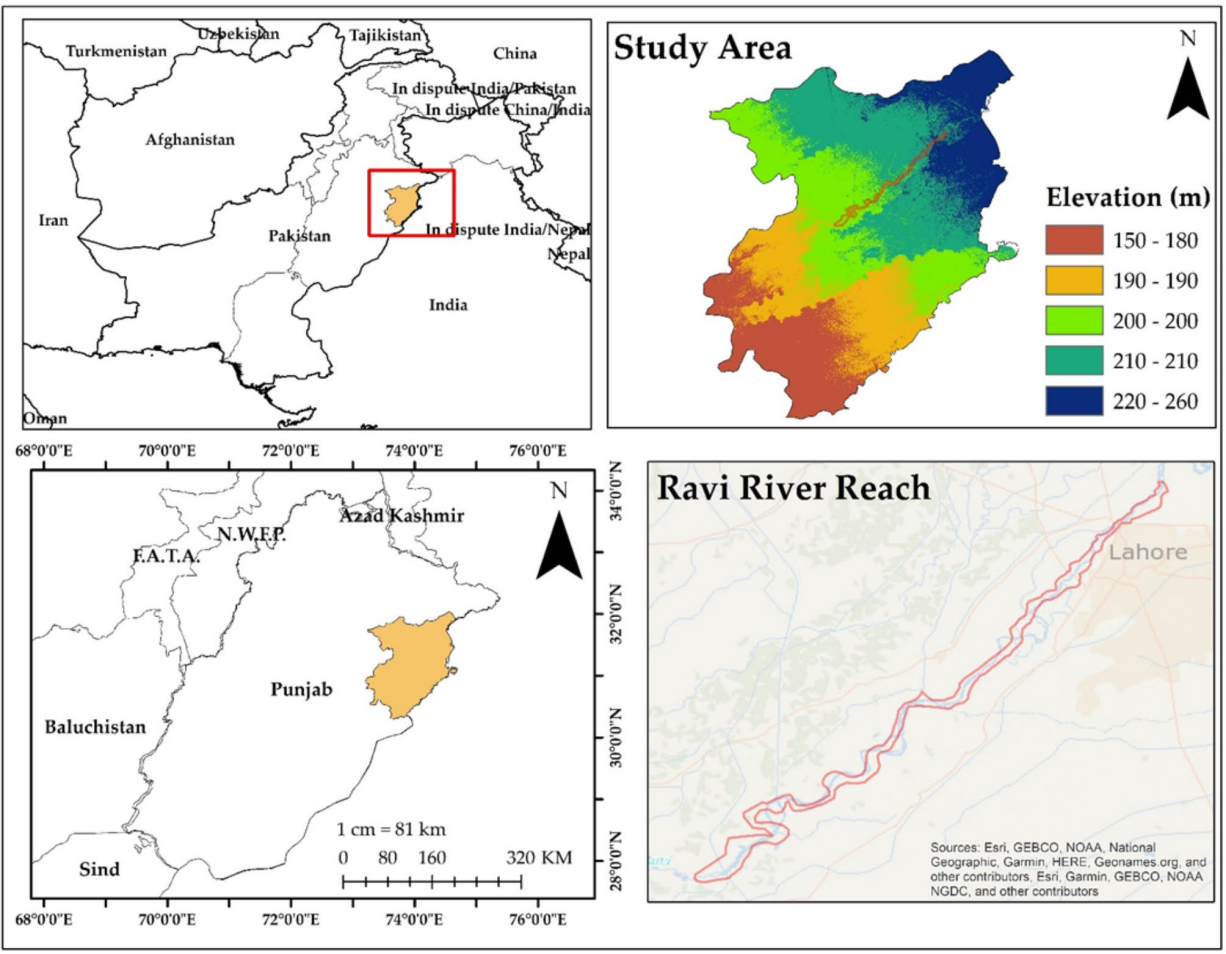
Map of the Study Area. Maps created using ArcGIS version 10.8.2^53^.

One of Pakistan’s most significant and rapidly expanding metropolitan hubs is Lahore. According to population statistics, the city district had 2,984,000 residents in 1981, 5,133,000 by 1998, and 8,091,000 by 2009. Lahore’s population has surpassed 13.09 million according to the 2021 census. A total of 13,095,000 people called Lahore’s metro region home in 2021, an increase of 3.58% from 2020. If a city’s population growth keeps up at the current rate, it will soon rank among the largest cities in the world. Lahore is expected to have 25 million residents by 2050. Lahore’s yearly average temperature is 35 °C, with monthly extremes of 22 °C in January and 45 °C in June. The climate in Lahore is classified as subtropical with a warm summer and pleasant winter. The city experiences 600 mm of yearly rainfall during the monsoon season, with July and August often having the highest amounts^50^, while the rest of the year is quite dry. Figure 1 shows a map of the study area.

**Fig. 2:**
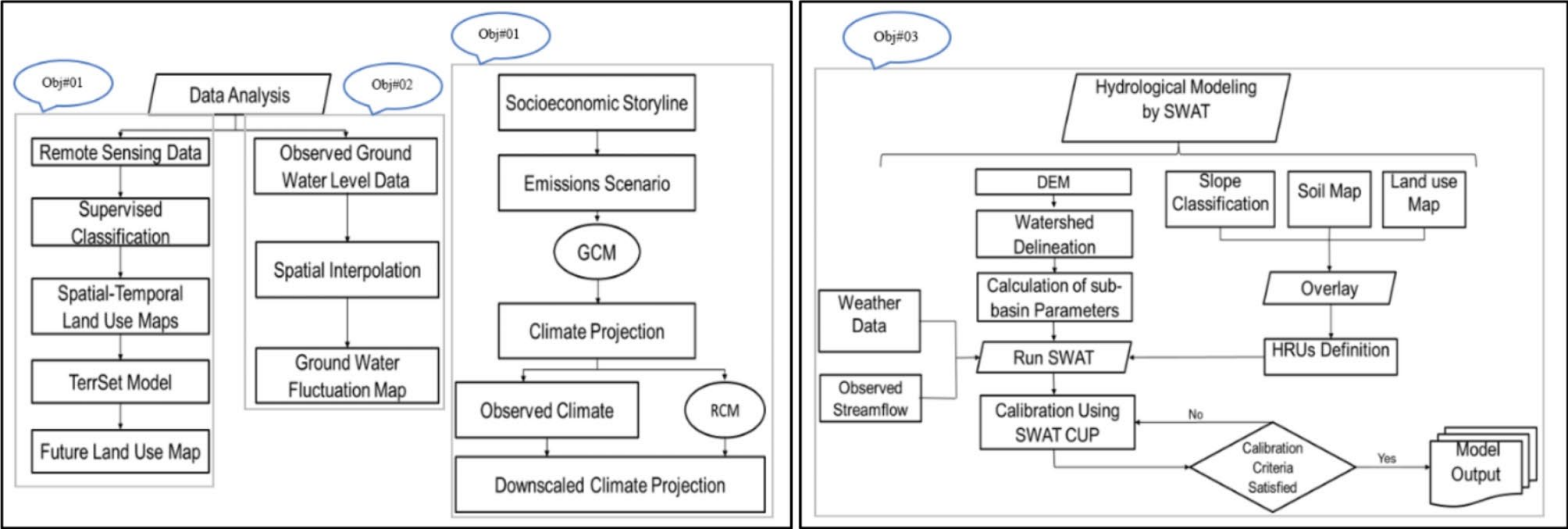
Methodology flowchart of the current study.

Every research project has a specific work schedule that must be adhered to from start to completion. Figure 2 gives the detailed methodology of the study. The method involves gathering, analyzing and extracting geographic information (GIS) characteristics and urban regions using SRTM 30 m DEM and Landsat imagery^51,52^. It also involves downscaling projected climate data using statistical methods and setting up a hydrological model, which entails preparing input data, calibrating and validating it.

### Data collection

A crucial component of scientific study is selecting appropriate data and acquiring that data from pertinent sources (Table 1). The meteorological data, including precipitation, temperature, relative humidity, sunshine duration, and wind speed data, were collected from the Pakistan Meteorological Department (PMD) from 1990 to 2020. Data on daily inflows at Balloki station for the period 1990–2020 were obtained from the Programme Monitoring & Implementation Unit (PMIU). The study area’s DEM with a 30 m resolution and Landsat images of the Tiff files of the study region were obtained from the USGS website.

**Table 1:** Description of sources of data collected.

Types	Data-Set	Source
Meteorological	Precipitation, Temperature, Sunshine Duration, Wind Speed (1990–2020)	Pakistan Meteorology Department (PMD) https://www.pmd.gov.pk/en/
Hydrological	Discharge (1990–2020)	Programme Monitoring & Implementation Unit (PMIU) & Irrigation Department https://irrigation.punjab.gov.pk/
Ground Water Level	Depth of Ground Water Tables (1996–2020)	Water & Sanitation Agency (WASA) https://wasa.punjab.gov.pk/
Topography	DEM (Spatial Resolution 30 m)	Shuttle Radar Topographic Mission (version 4) https://earthexplorer.usgs.gov/
Landcover	Landsat (4–5,8) Images (Spatial Resolution 30 m)	USGS https://earthexplorer.usgs.gov/
CMIP6 Model data	MPI-ESM1-2-HR (Nominal Resolution 100 km) (2020–2100)	Coupled model Intercomparison Project (CMIP) https://wcrp-cmip.org/cmip-data-access/

### GCM data downscaling

Global climate models (GCMs) are intricate mathematical representations of the principal parts of climate systems and how they communicate^53–58^. The world’s air into network boxes frequently has an accuracy of 100–200 km. On every matrix, conditions portraying barometrical elements are addressed. Because the surface geology is additionally controlled at similar spatial scales (100–200 km), a few critical actual cycles and meteorological phenomena cannot be precisely addressed. Consequently, downscaling of the GCM-simulated data uses the data for study at finer scales. Downscaling transforms large-scale data to small-scale data (i.e., global climate data to regional climate data). New information is introduced into GCM outputs based on observed meteorological data or high-resolution modeling of physical processes. In this study, statistical downscaling simulated by the GCM (MPI-ESM1-2-HR) maximum and minimum temperature and precipitation data was performed using the CMhyd (Climate Model data for hydrologic modeling) model. Previous studies chose MPI-ESM1-2-HR due to its easily accessible data, high resolution, and proven effectiveness at the basin scale^10^. Statistical downscaling is a process used to estimate local or regional climate variables based on large-scale climate information.

### Climate downscaling model (CMhyd)

Using the CMhyd model, bias correction at the river scale was performed to address predicted biases in temperature and precipitation from the MPI-ESM1-2-HR model under SSP2 and SSP5 events^59,60^. Global adoption of the CMhyd model has shown promise in filling the data gap between generated information from GCMs and real climate factors from sensors^2^. Haider et al.^2^ show the downsizing of GCM results in the river basin size may be done confidently using the CMhyd model. It provides different statistical methods of downscaling precipitation and temperature. The GCM results for the 2020–2100 timeframe were downscaled in this work using SSP2 and SSP5 scenarios. This required combining daily precipitation, maximum temperature, and lowest temperature values gathered from 1990 to 2020 as a foundation with forecast predictions from GCM.

### Land use change analysis

For the intended areas for the years 1995, 2000, 2010, 2015, 2018, and 2020, we obtained free Landsat images. Two distinct Landsat image tiles covered the research area. Therefore, for each of the aforementioned years, these four tiles, which cover the catchment, were downloaded. These tiff-format tiles were stacked using the Erdas Imagine software. Following the stacking procedure in the image processing program, the Landsat tiles were mosaicked to produce a single image. The catchment was extracted once the downloaded tiles had been mosaicked. This process was performed with the mosaicking tool in ArcGIS utilizing the program’s bulk feature extraction function. The same method was used for the Landsat images from 1995, 2000, 2010, 2015, 2018, and 2020.

The preparation of land cover maps for the present and earlier years included the utilization of regulated picture order. The downloaded Landsat images were handled in ArcGIS before being identified using the maximum probability method-the most used supervised image classification method. The combinations of bands and their attributes are shown in Table 2.

**Table 2:** Landsat Band combinations for information extraction.

Visible attribute	Band combination
Color Infrared (vegetation)	5 4 3
False Color (urban)	7 6 4
Natural Color	4 3 2
Agriculture	6 5 2
Shortwave Infrared	7 5 4
Healthy Vegetation	5 6 2
Land/Water	5 6 4

### Future land use change analysis

**Fig. 3:**
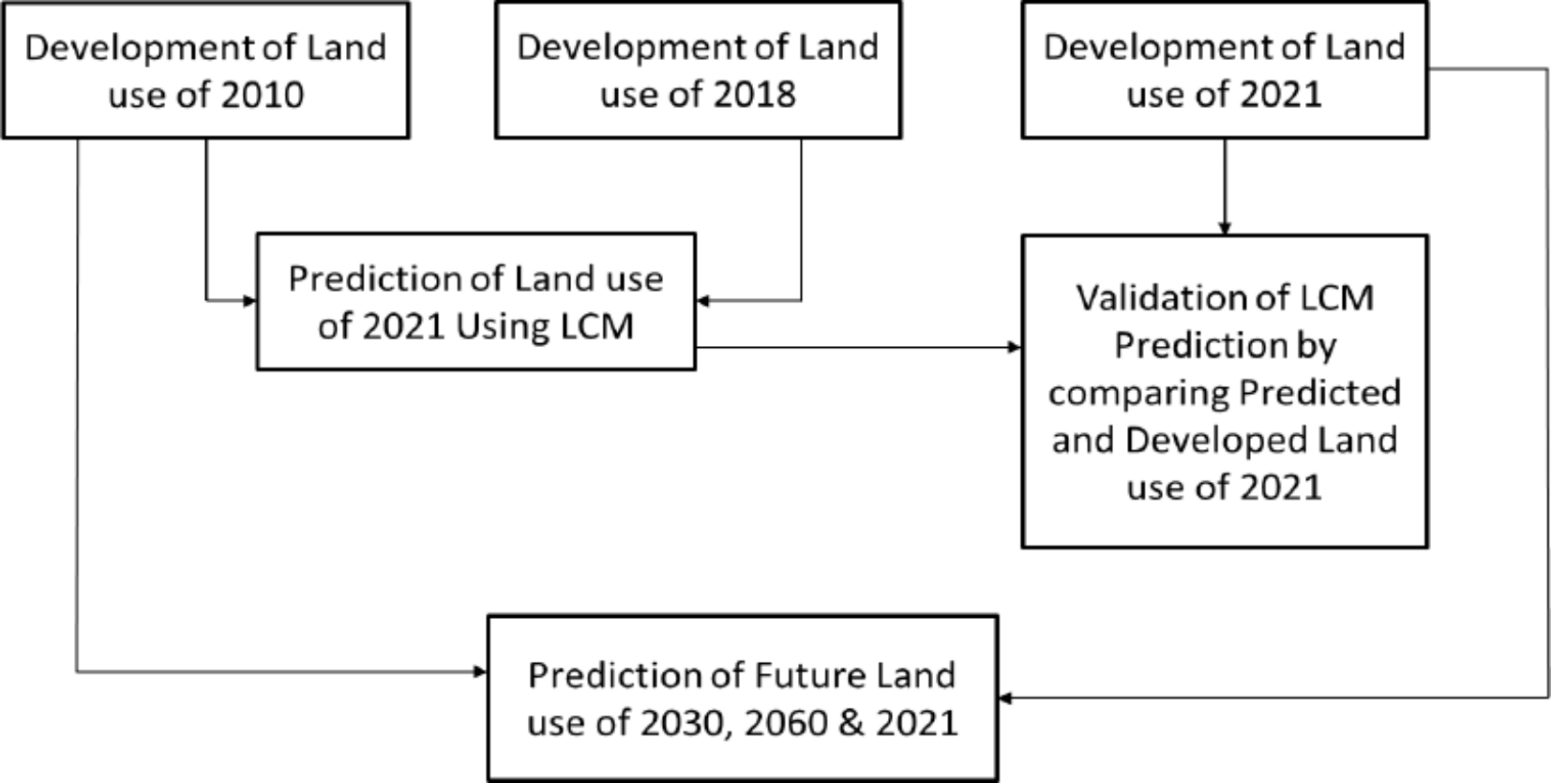
Future land use preparation approach.

Future land use change was predicted using the TerrSet CA (Cellular Automata) Markov model (Fig. 3). The CA-Markov module was used to simulate various combinations of multiple-category LUCAs^61–67^. This approach facilitates the creation of a matrix that compares the transitions between earlier land-use maps and calculates the likelihood that these transitions occur in future land-use change predictions^68–70^. A flowchart of the future land use methodology is presented in Fig. 3.

### Analysis of Markov chain (MCA) investigation

Markov Chain Analysis (MCA) is a widely applicable tool for predicting changes, known for its ability to manage large-scale and unpredictable dynamics. By utilizing historical data, it makes predictions about future trends. MCA can be employed when an area is divided into cells, with each cell transitioning from one land cover (LC) type to another over a specific period. At any given moment, each cell will exhibit a particular LC type. The transition probability measures the likelihood of a condition changing. The conversion grid generated by MCA includes the expected visual changes and the probability of a certain LC category transitioning to a different one^71,72^. The Markov transition matrix P is represented by Eq. 1:

1 $$\left\| {{{\text{P}}_{{\text{ij}}}}} \right\| = \left\| {\begin{array}{llll} {{{\text{P}}_{1,1}}} & {{{\text{P}}_{2,1}}} & { \ldots \ldots \ldots } & {{{\text{P}}_{{\text{N}},1}}} \\ {{{\text{P}}_{1,1}}} & {{{\text{P}}_{2,2}}} & { \ldots \ldots \ldots } & {{{\text{P}}_{{\text{N}},2}}} \\ {{{\text{P}}_{1,1}}} & {{{\text{P}}_{2,3}}} & { \ldots \ldots \ldots } & {{{\text{P}}_{{\text{N}},3}}} \\ \cdot & \cdot & \cdot & \cdot \\ \cdot & \cdot & \cdot & \cdot \\ \cdot & \cdot & \cdot & \cdot \\ {{{\text{P}}_{{\text{N}} - 1,1}}} & {{{\text{P}}_{{\text{N}} - 2,2}}} & { \ldots \ldots \ldots } & {{{\text{P}}_{{\text{N}} - 1,{\text{N}}}}} \\ {{{\text{P}}_{{\text{N}},1}}} & {{{\text{P}}_{{\text{N}},2}}} & { \ldots \ldots \ldots } & {{{\text{P}}_{{\text{N}},{\text{N}}}}} \\ \end{array}} \right\| \quad \left( {0 \le {{\text{P}}_{{\text{ij}}}} \le 1} \right)$$

Where $\:{\text{P}}_{\text{i}\text{j}}$​ represents the transition probability from land cover type 1 to land cover type 2 over the first and second periods of land cover. Following several predefined periods, a set of conditioned probabilistic images is produced, commonly used for transient possible images. These graphics depict the likelihood of each type of land cover occurring in all images. In a Markov analysis, the causes of land cover change (LCC) are not considered. Since Markov analysis lacks spatial sensitivity, it can significantly impact the environment. Therefore, Cellular Automata (CA) provides a geographic dimension to the modeling approach.

### Cellular automata (CA)-Markov

The TERRSET library’s CA and CA_MARKOV components were utilized to forecast prospective land cover trends. To increase geographic precision and predict changes in worldwide distribution, the CA MARKOV framework combines Markov chains, Cellular Automata (CA), Multi-Objective Land Allocation (MOLA), and multicriteria techniques. This technique uses two historical maps of land covering and Markov Chain Analysis (MCA). To estimate the amount of land cover changes from current classes to other classes throughout the projected time, transition zone data is used. The initial land cover imagery is used to start the alteration training, and later land cover images are used for the MCA^66^. Maps with change potential are used to assess how well suited each pixel is for various forms of land cover. Next, a contiguity filter lowers the weight of pixels farther away from these zones and prioritizes adjacent permitted zones.

### River network and watershed delineation

A DEM was used for preparing the river network, demarcating the watershed, evaluating the catchment slope, and determining the length and timing of the concentration. The river network for the Ravi River at Balloki was constructed using ArcGIS software version 10.5 with Arc Hydro tools. The DEM file of the research area was initially transformed from a geographic coordinate system to a UTM. Then, various stages, including fill sink definition, stream amassing, stream definition, stream division, catchment network depiction, catchment polygon handling, and seepage line handling, were performed. These steps are all described in ArcHydro tools.

After the construction of river networks, watersheds are formed by seepage point handling. Watershed handling occurs at that point, and the bunch point or point inclusion for the watershed’s flight can be determined by providing its scope and longitude. The River Ravi’s watershed was established around the outflow point after the outlet point was covered^73–75^.

**Fig. 4:**
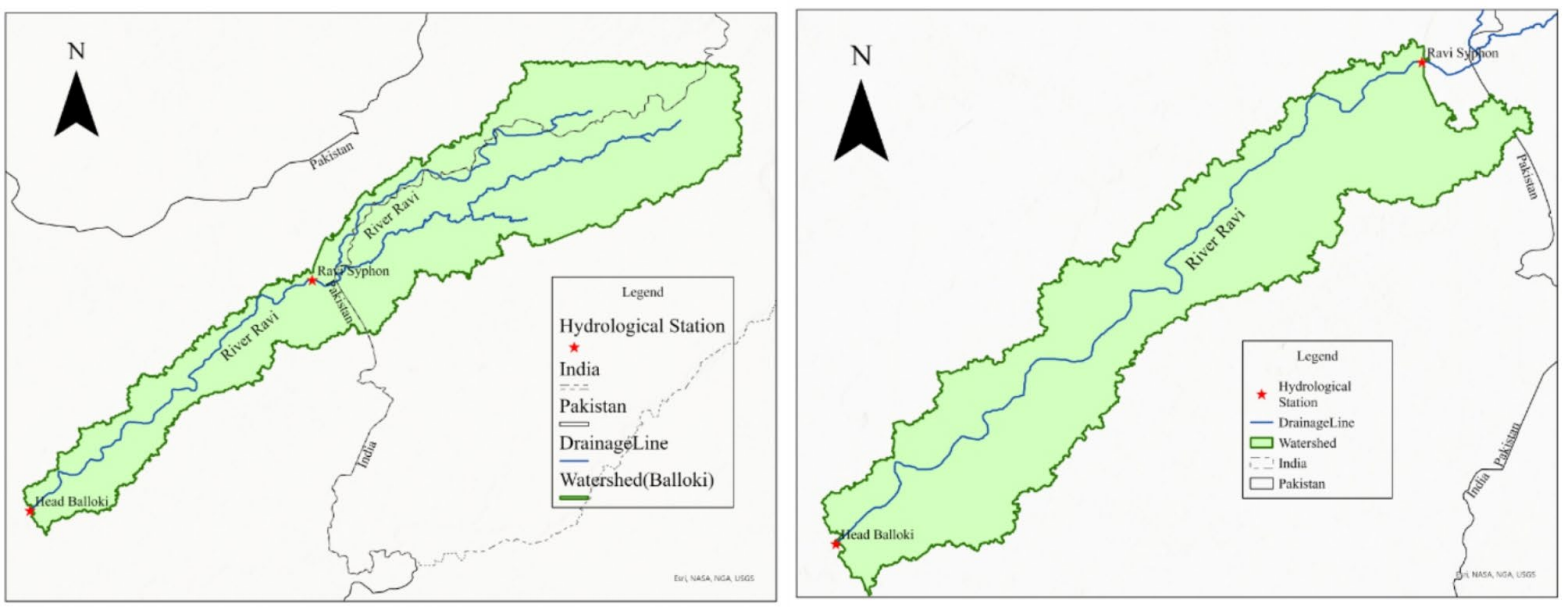
Watershed of the Ravi River from Ravi Syphon to Balloki Headworks. Maps created using ArcGIS version 10.8.2 ^53^.

The flows of the Ravi River enter Pakistan from India. Figure 4 shows the upper part of India and the lower part of Pakistan. The working area was the Balloki Headword to Ravi Syphon, and the upper portion was subtracted. In this work, hydrological modeling of the Ravi River near Balloki was carried out for 1990–2100 to assess the impacts of climate and land use on the water supplies in the Balloki region. The discharge data of the Upper Chenab Link Canal and Qadirabad Balloki Link Canal were subtracted from the Ravi Syphon discharge data.

### Hydrological modeling

The SWAT model is a widely used tool for simulating various hydrological processes, including streamflow, in watershed systems^76^. This model is considered superior for assessing climate change and land use impacts due to its comprehensive and flexible nature, which allows for detailed simulation of various hydrological processes across different spatial and temporal scales. It integrates climate data, land use, soil properties, and water management practices, providing a holistic view of watershed dynamics. One of its primary advantages is its ability to model large, complex watersheds with diverse land uses and climatic conditions, making it highly applicable for long-term impact studies^2,77^. SWAT’s open-source nature and extensive user community also facilitate continuous improvements and support. However, its complexity can be a disadvantage, requiring significant expertise and computational resources to set up and run simulations accurately. Calibration and validation can also be time-consuming and data-intensive, potentially limiting its use in data-scarce regions. Its application aids in water resource management, flood forecasting, and assessing the impact of land use changes on streamflow dynamics in the region^78^. The basic equation used in the SWAT model is shown below.

2 $$\:{\text{S}\text{W}}_{\text{t}}={\text{S}\text{W}}_{0}+{\sum\:}_{\text{i}=1}^{\text{t}}\left({\text{P}}_{\text{d}\text{a}\text{y}}-{\text{Q}}_{\text{s}\text{u}\text{r}\text{f}}-{\text{E}}_{\text{a}}-{\text{W}}_{\text{s}\text{e}\text{e}\text{p}}-{\text{Q}}_{\text{g}\text{w}}\right)$$

Where $\:{\text{S}\text{W}}_{\text{t}}$ is the final soil water (mm), $\:{\text{S}\text{W}}_{0}$ is the initial soil water (mm), $\:{\text{P}}_{\text{d}\text{a}\text{y}}$ is the precipitation at time i (mm), $\:{\text{Q}}_{\text{S}\text{u}\text{r}\text{f}}$ is the surface runoff (mm), $\:{\text{E}}_{\text{a}}$ is the evapotranspiration (mm), $\:{\text{W}}_{\text{s}\text{e}\text{e}\text{p}}$ is the water flow to the unsaturated zone (mm), and $\:{\text{Q}}_{\text{g}\text{w}}$ is the water flow of the watershed from underground (mm).

To acquire the best values for sensitive parameters, model calibration was performed. Then, SWAT-CUP, an auto calibrating tool, was employed. The autocalibration feature present in SWAT 2009 is not available in SWAT 2012^79^. Therefore, SWAT-CUP is now used for autocalibration. The calibration was performed using streamflow measurements in monthly time steps at Balloki between 1999 and 2002. The shuffled complex evolution algorithm (SCE-UA) and a single goal function form the foundation of the calibration process^29,80^. A multi objective, automated calibration process that was created by Karim C. Abbaspour called SUFI-2 was employed in this study. This approach was chosen because it may be used with straightforward and intricate hydrological models. The intended optimization parameters, the observed data file, and the calibration techniques were subsequently chosen. As a result, many flow parameters were considered throughout the calibration procedure. After the automatic calibration, SWAT was run with the best parameter values. The selected calibration and validation parameters are shown in Table 3.

**Table 3:** Calibration and validation parameters selected for Hydrological modeling.

Categories	Parameters	Parameters Detail
Groundwater Parameters	REVAPMN.gw	Threshold depth of water in the shallow aquifer for “revap” to occur (mm)
	GW_DELAY.gw	Groundwater delay (days)
	GWQMN.gw	Threshold depth of water in the shallow aquifer required for return flow to occur (mm)
Hydrologic Response Unit Parameters	ESCO.hru	Soil evaporation compensation factor
	HRU_SLP.hru	Average slope steepness
	SLSUBBSN.hru	Average slope length
	OV_N.hru	Manning’s “n” value for overland flow
	CANMX.hru	Maximum canopy storage
Routing Parameters	CH_K2.rte	Effective hydraulic conductivity in main channel alluvium
	CH_N2.rte	Manning’s “n” value for the main channel
Watershed Parameters	SURLAG.bsn	Surface runoff lag time
Management Parameters	CN2.mgt	SCS runoff curve number
Soil Parameters	SOL_BD(.).sol	Moist bulk density
	SOL_K(.).sol	Saturated hydraulic conductivity
	SOL_AWC(.).sol	Available water capacity of the soil layer
Sub-Basin Parameters	CH_N1.sub	Manning’s “n” value for the tributary channels

### Performance indicators

The statistical analysis section involved the calibration and validation of the hydrological model using key performance metrics including root mean square error (RMSE), the Nash-Sutcliffe efficiency (NSE), and the coefficient of determination (R^2^). During the calibration phase, the model demonstrated a satisfactory fit with observed data, yielding an RMSE value indicating the average discrepancy between simulated and observed values. Additionally, the NSE coefficient, measuring the relative magnitude of the residual variance compared to the observed data variance, indicated a high degree of model efficiency. The R^2^ coefficient, representing the proportion of the variance in the observed data that is predictable by the model, further confirmed the model’s robustness in replicating the observed hydrological responses. Subsequent validation of the model for an independent dataset reinforced these findings, with RMSE, NSE, and R^2^ values indicating a consistent performance^81–88^, thus bolstering confidence in the model’s reliability for simulating the hydrological behavior of the study area.

#### Coefficient of determination (R2)

R^2^ measures the distance of the data to the fitted regression line. The mathematical expression for R² is shown in Eq. 3:

3 $$\:{\text{R}}^{2}=\frac{\text{N}\sum\:{\text{Q}}_{\text{o}}{\text{Q}}_{\text{m}}-\sum\:{\text{Q}}_{\text{o}}\sum\:{\text{Q}}_{\text{m}}}{\sqrt{\left[\text{N}\sum\:{{\text{Q}}_{\text{o}}}^{2}-{\left(\sum\:{\text{Q}}_{\text{o}}\right)}^{2}\right]\left[\sum\:{{\text{Q}}_{\text{m}}}^{2}-{\left(\sum\:{\text{Q}}_{\text{m}}\right)}^{2}\right]}}$$

where N is the number of observations, Q_o_ is the observed discharge value, and Q_m_ is the modeled discharge value.

The value of R² ranges between 0 and 1. A value of zero indicates that the model failed to explain the data variability around the average. One indicates that the model successfully explained all the variability in the data around its average. Generally, the closer the R² values are to 1, the better the fit of the model^81–88^.

#### Nash-Sutcliffe efficiency (NSE)

The Nash Sutcliffe coefficient is the hydrological model efficiency coefficient, and its value indicates how good the model prediction is. The mathematical expression (4) for the Ns coefficient is:

4 $$\:\text{N}\text{S}\text{E}=1-\:\frac{\sum\:_{\text{i}=1}^{\text{N}}{\left({\text{Q}}_{\text{o}}-{\text{Q}}_{\text{m}}\right)}^{2}}{\sum\:_{\text{i}=1}^{\text{N}}{\left({\text{Q}}_{\text{o}}-\stackrel{-}{{\text{Q}}_{\text{o}}}\right)}^{2}}$$

where N is the number of observations, Q_o_ is the observed discharge value, Q_m_ is the modeled discharge value and $\:\stackrel{-}{{Q}_{o}}$ is the average observed discharge value.

Its values range from - ∞ to 1. When the NSE = 1, the modeled discharge best matches the observed values. When NSE = 0 is used, the observed values’ average matches the modeled predictions’ accuracy. When NSE ≤ 1 or a negative value occurs, the model’s predictions do not match the average of the observed values, and the mean of the observed values is a better predictor. The closer the value of Ns is to 1, the more accurate the model is^81–88^.

#### Root mean square error (RMSE)

The root mean square error is another statistical parameter used to determine the difference between modeled and observed values (Eq. 5). The standard deviation of the difference between the observed data and corresponding predicted/modeled values is shown. It is simple to test the accuracy of model predictions and to evaluate the prediction errors of different models. The method aggregates the magnitude of errors in modeled values for various calculations into a single measure of predictive power^81–88^. This parameter is sensitive to outliers.

5 $$\:\text{R}\text{M}\text{S}\text{E}=\sqrt{\frac{1}{\text{N}}\sum\:_{\text{i}=1}^{\text{N}}{\left({\text{Q}}_{\text{o}}-{\text{Q}}_{\text{m}}\right)}^{2}}$$

### Groundwater depth

**Fig. 5:**
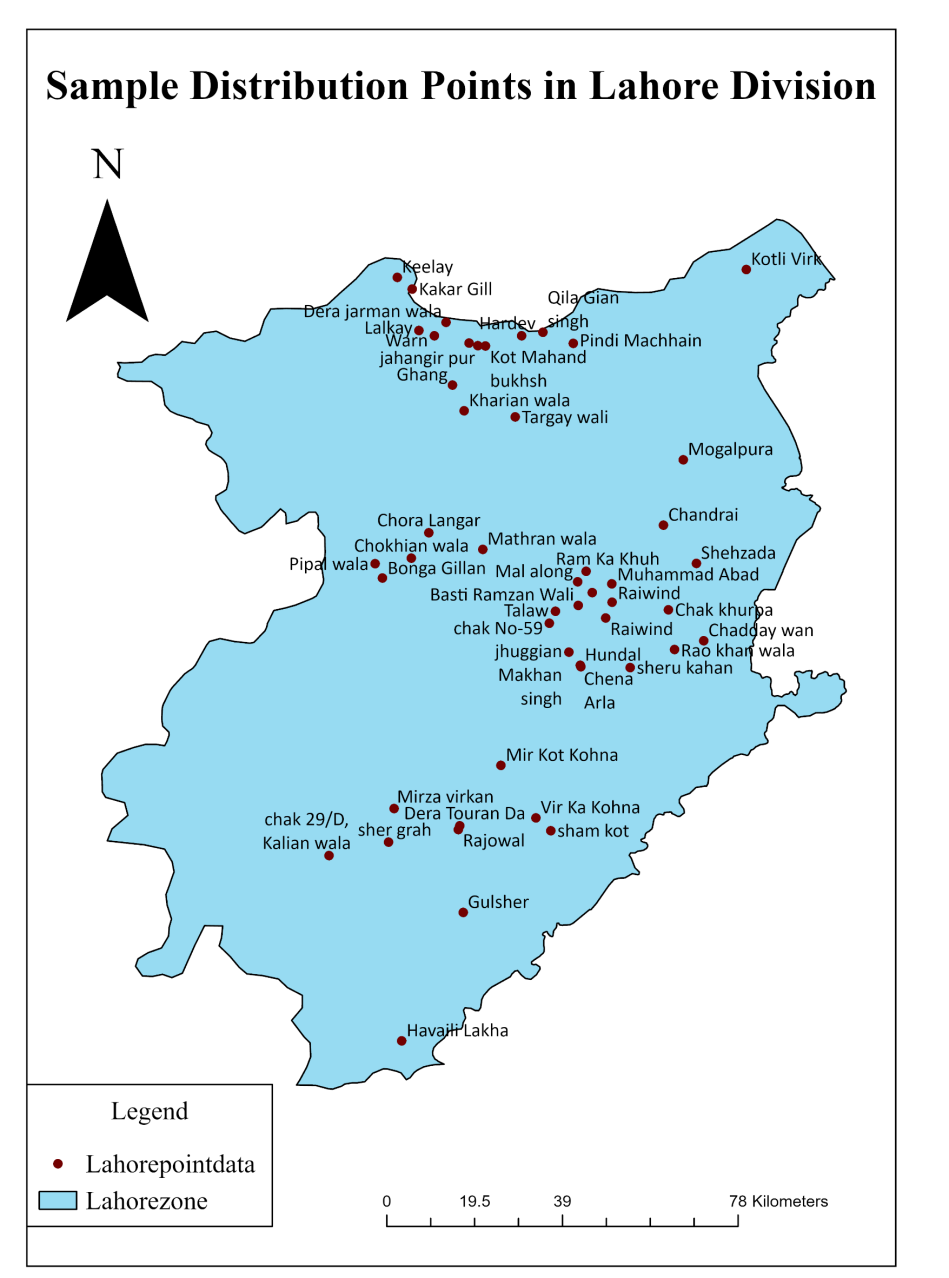
Distribution of past observed groundwater level points. Maps created using ArcGIS version 10.8.2 ^53^.

Figure 5 shows the groundwater depth data (pre- and post-monsoon) collected from the Lahore Irrigation and WASA Departments for 25 years (1996–2020) to create temporal groundwater maps for the city district of Lahore. The data were subsequently cleaned by removing the dead points on the map showing values of zero ‘0 m’, and the remaining points were plotted on the Lahore shape file in ArcGIS software. The collected water table data from 1996 to 2020 were then stored in the geodatabase and processed using ArcGIS software version 10.5. The observed ground water level point distributions are presented in Fig. 5.

The interpolation technique, inverse distance weighting (IDW), was used to estimate the spatial fluctuations in groundwater quality over the study area. IDW is a method of interpolation that estimates cell values by averaging the values of sample data points in the neighborhood of each processing cell. The closer a point is to the center of the cell being estimated, the more influence, or weight, it has in the averaging process. This method assumes that the mapped variable decreases influence with distance from its sampled location.

## Results

### Climate change projections

#### Projections of precipitation

**Fig. 6:**
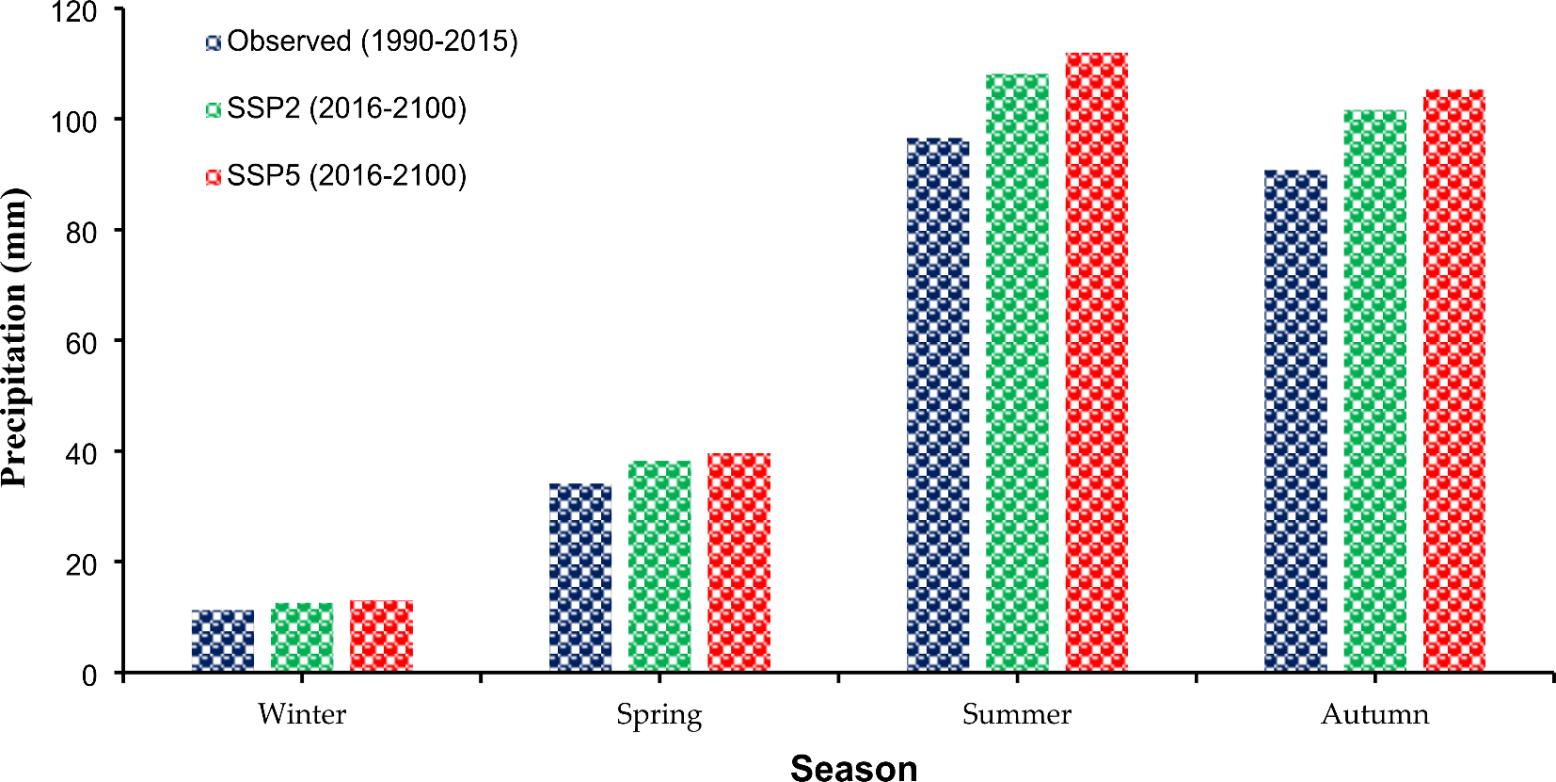
Comparison of Seasonal Precipitation at Lahore under Climate Change Scenarios SSP2 and SSP5.

The seasonal variation in precipitation reveals an increase in all seasons. This was presented by dividing the months of the year into four classes: winter (November, December, and January), spring (February, March, and April), summer (May, June, and July), and fall (August, September, and October). Winter precipitation increased under SSP2 and SSP5 from 11.26 mm to 12.61 mm and 13.06 mm, respectively (Fig. 6). Precipitation in the late spring and autumn pursued a comparative direction but with a more sensitive increase, from 96.54 mm to 108.12 mm and 112 mm under SSP2 and SSP5, respectively, in the mid-year and from 90.75 mm to 101.64 mm and 105.3 mm under SSP2 and SSP5, respectively, in the autumn season. Similarly, under SSP2 and SSP5, the springtime precipitation in Lahore is expected to increase from 34.11 mm to 38.21 mm and 39.57 mm, respectively^1,4,89^.

#### Projections of maximum temperature

**Fig. 7:**
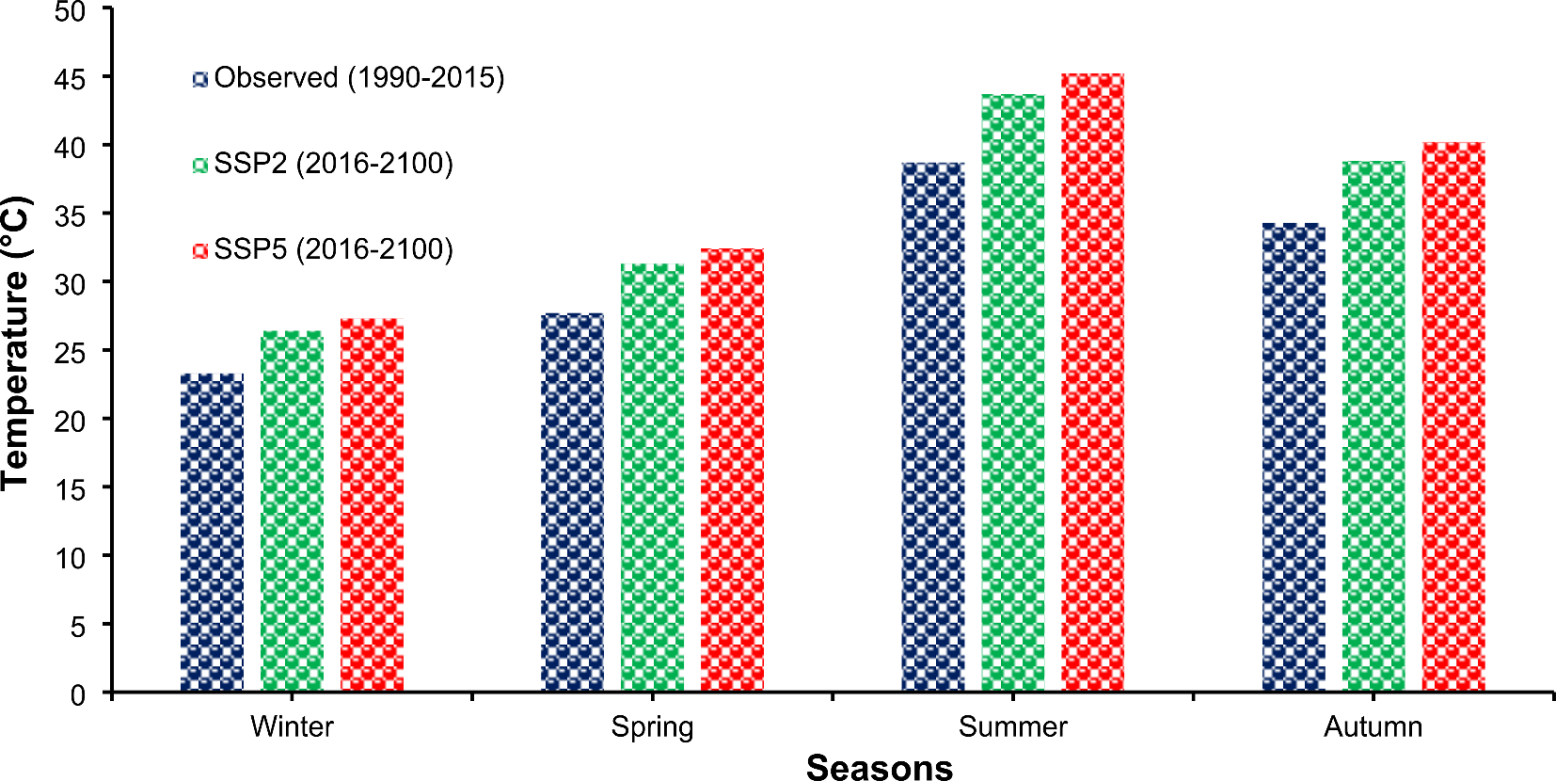
Comparison of Seasonal Maximum Temperatures at Lahore under Climate Change Scenarios SSP2 and SSP5.

Examining the temperature changes revealed that the maximum temperature expanded across the four seasons. This modification involved dividing the months of the year into four classes: winter (November, December, and January), spring (February, March, and April), summer (May, June, and July), and autumn (August, September, and October). As shown in Fig. 7, under SSP2 and SSP5, during the winter of the year, the maximum temperature climbed from 23.3 °C to 26.4 °C and 27.3 °C, respectively. The summer maximum temperatures increase from 38.7 °C to 43.7 °C and 45.2 °C under SSP2 and SSP5, respectively; the spring maximum temperatures increase from 27.7 °C to 31.3 °C and 32.4 °C under SSP2 and SSP5, respectively; and the autumn maximum temperatures increase from 34.3 °C to 38.8 °C and 40.2 °C under SSP2 and SSP5, respectively. Haider et al.^2^ reported that the maximum temperature increased to 43.7 °C and 45.2 °C under the SSP2 and SSP5 scenarios, respectively. Similarly, a study performed by Masood et al.^10^ showed that the maximum temperature will increase until the end of the 21st century.

#### Projections of the minimum temperature

**Fig. 8:**
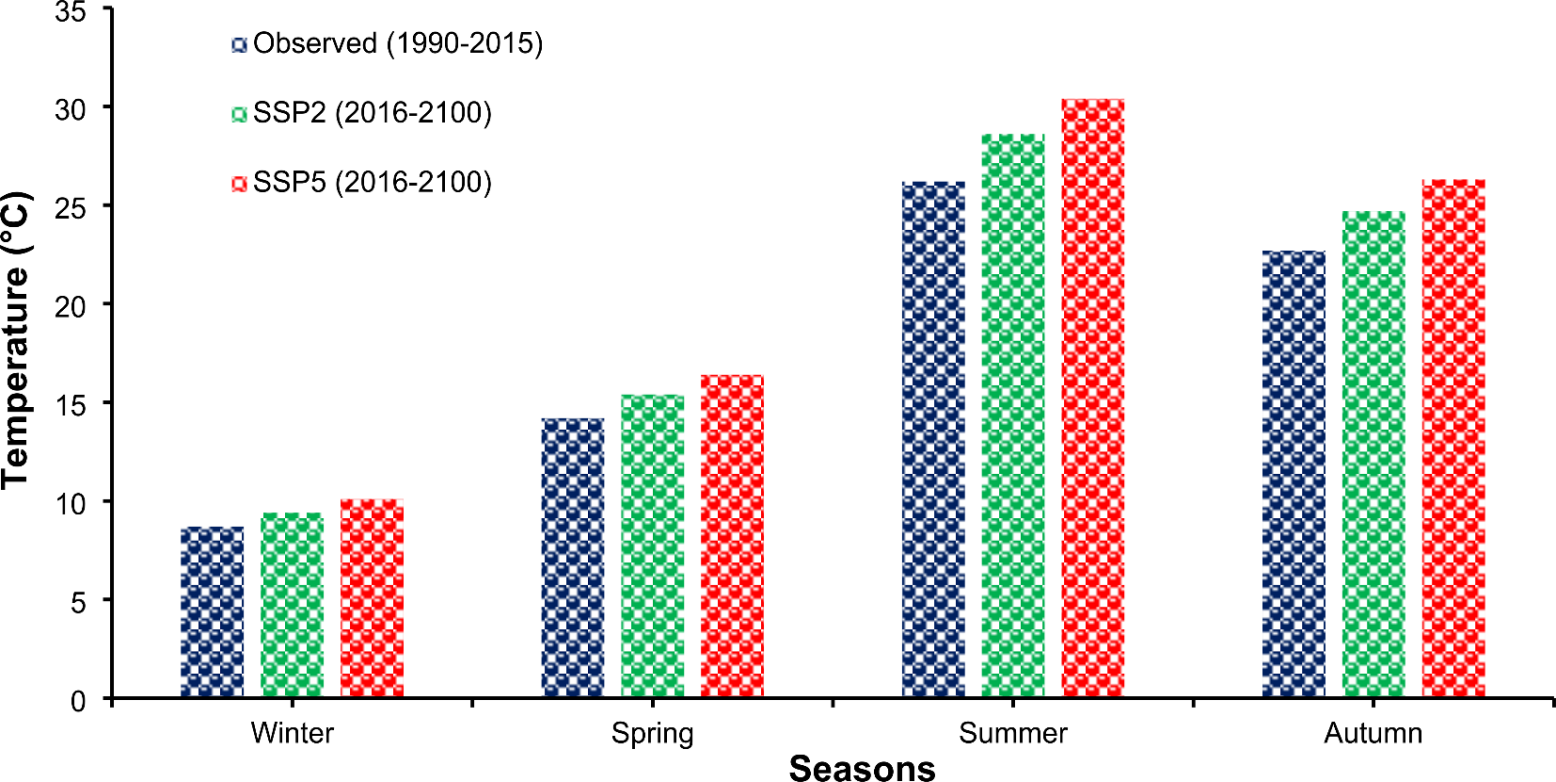
Comparison of Seasonal Minimum Temperatures at Lahore under Climate Change Scenarios SSP2 and SSP5.

Examining temperature changes revealed that the minimum temperature increased across the year’s four seasons. As shown in Fig. 8, under SSP2 and SSP5, the wintertime minimum temperature climbed from 8.7 °C to 9.4 °C and 10.1 °C, respectively. The summer minimum temperature increased from 26.2 to 28.6 °C under SSP2 and 30.4 °C under SSP5, while the autumn minimum temperature increased from 22.7 to 24.7 and 26.3 °C under SSP2 and SSP5. Moreover, the minimum temperature of spring increased from 22.7 °C to 24.7 °C and 26.3 °C under SSP2 and SSP5, respectively Haider et al.^2^, and the minimum temperature increased to 24.7 °C and 26.3 °C under SSP2 and SSP5, respectively. A study conducted by Anjum et al. 2019 revealed that temperature will increase under future scenarios.

### Land use dynamics

**Fig. 9:**
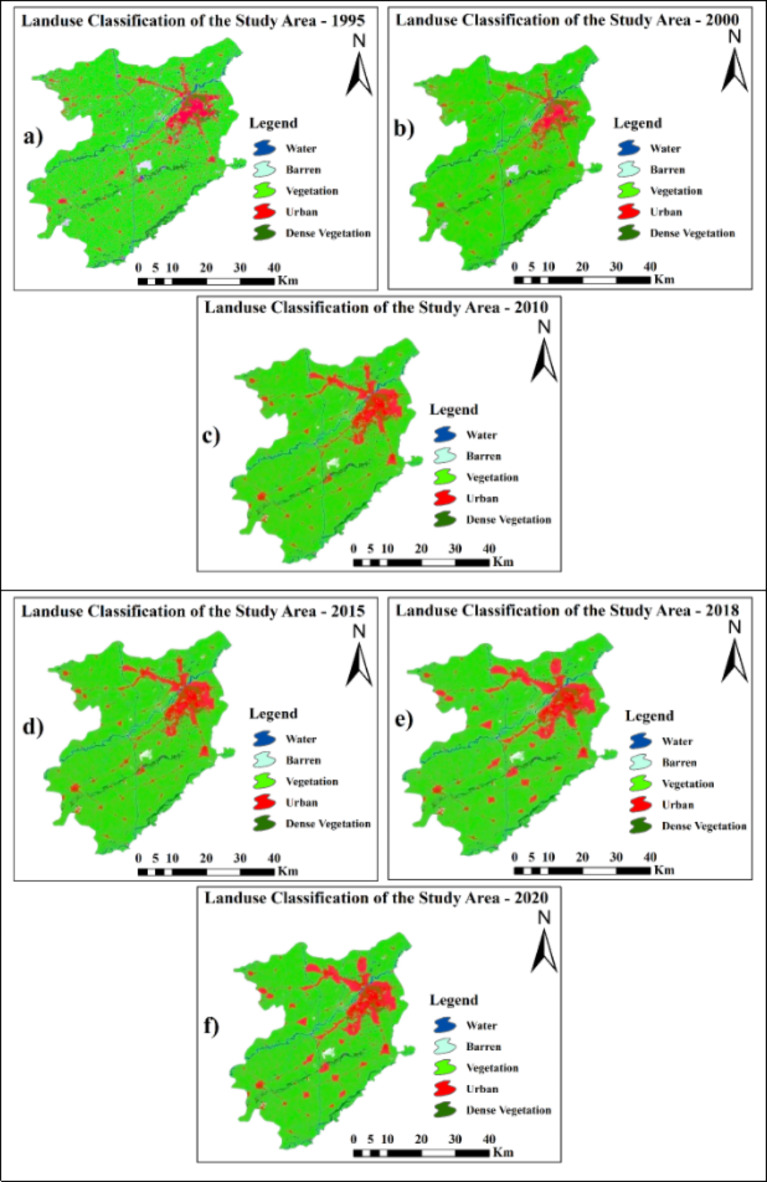
Land use maps of different years from 1995 to 2020. Maps created using ArcGIS version 10.8.2^53^.

**Fig. 10:**
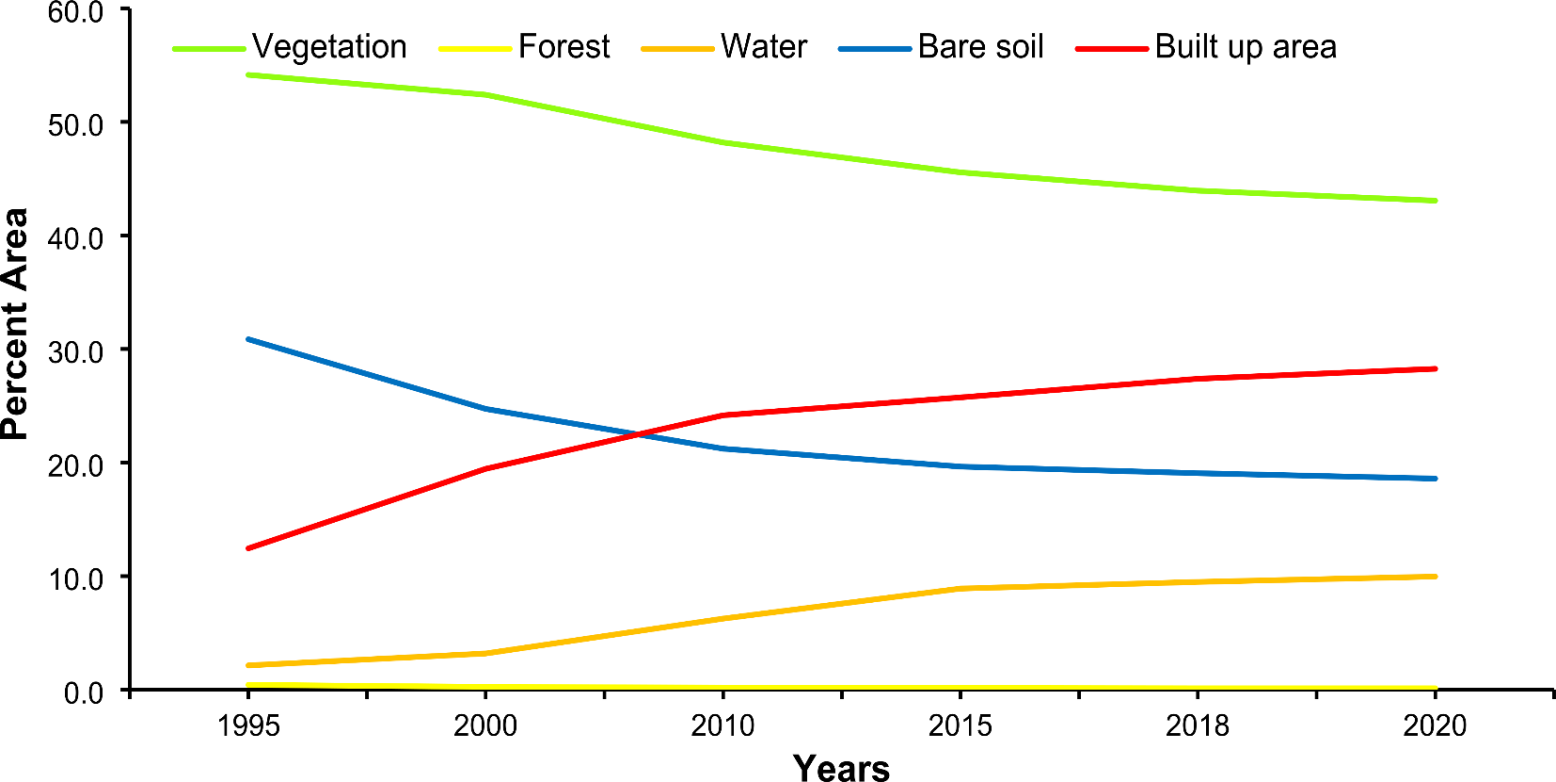
Trends in Land Use Change from 1995 to 2020.

Figure 9 classified land use maps for 1995, 2000, 2010, 2015, 2018, and 2020 are shown for the study area. Five categories—water, bare soil, vegetation, and built-up area—were used to construct the land use map. The analysis revealed that from 1995 to 2020, the vegetation, forest, and bare soil coverage decreased by 11.1%, 0.3%, and 12.3%, respectively. This has led to growth in the built-up area and water surface area by 15.8% and 7.8%, respectively. Figure 10 shows this shift in land use classes.

### Future land use maps

**Fig. 11:**
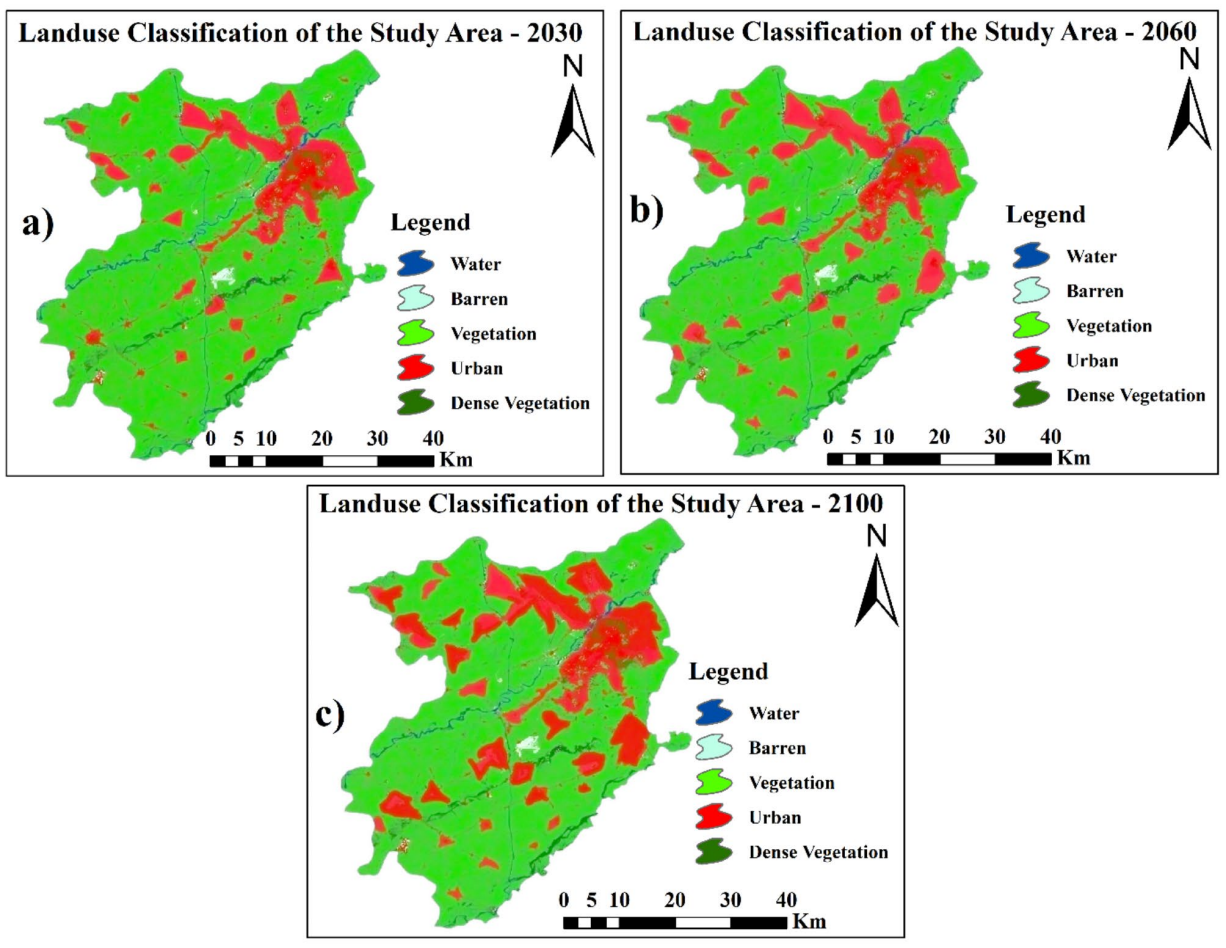
Future land use maps for 2030, 2060, and 2100. Maps created using ArcGIS version 10.8.2 ^53^.

**Fig. 12:**
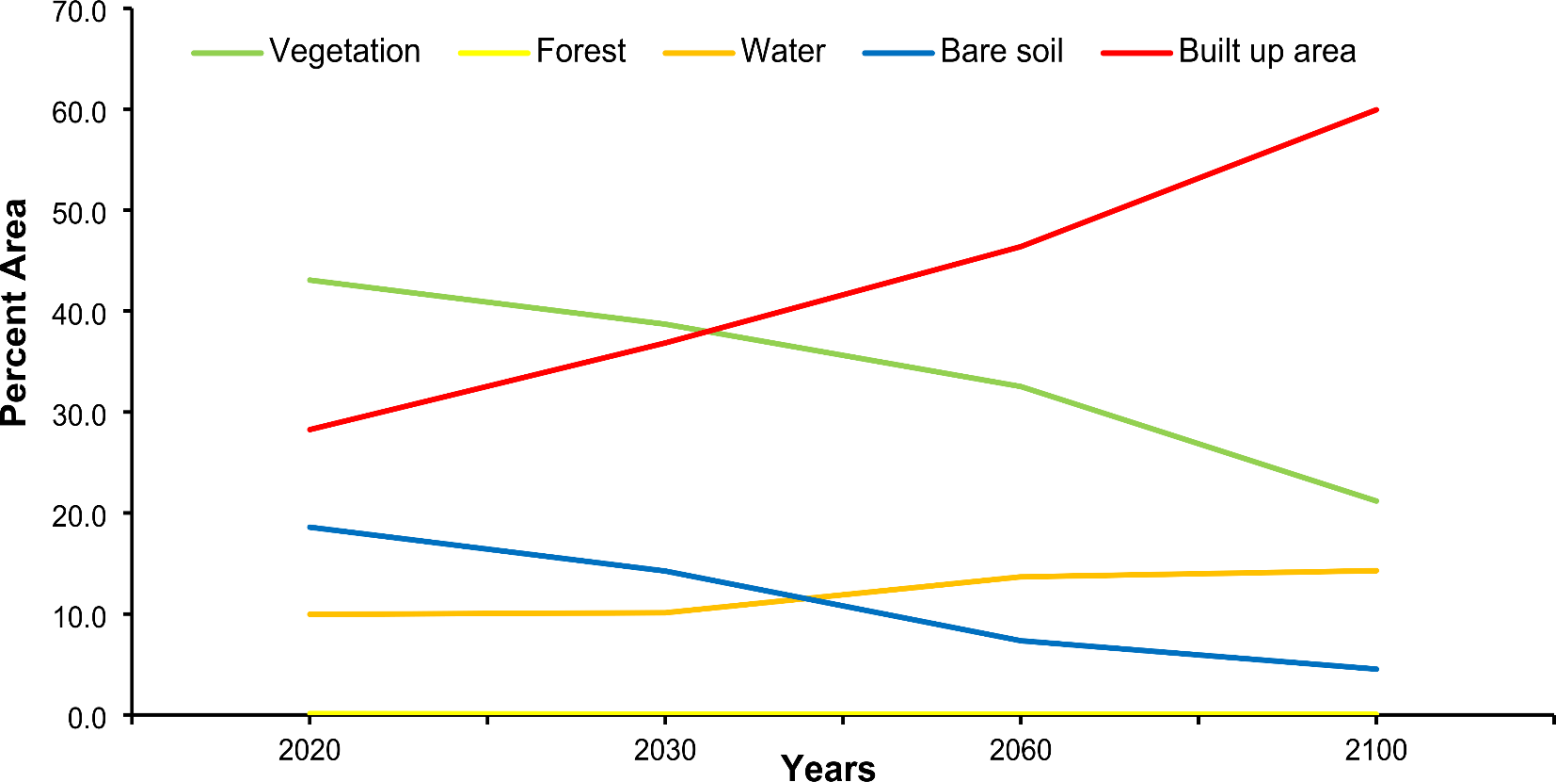
Trends in Land Use Change from 2020 to 2100.

With the embedded Land Change Modeler in TerrSet, land cover maps for the future (2030, 2060, and 2100) were generated. TerrSet, a Geospatial Monitoring and Modeling System featuring tools for analyzing image time series, was developed in 1987 by Prof. J. Ronald from Clark University. Future land cover simulations will utilize the cellular automata-Markov chain model (CA-MCM). In Fig. 11, classified maps of the study area are presented. According to these findings, Lahore’s built-up area and water body area will increase by 31.7% and 4.3%, respectively, between 2020 and 2100. Lahore’s built-up area has expanded over time due to industry and population growth. As more land is developed for residential, commercial, and industrial purposes, the cityscape expands outward. This urban sprawl converts natural landscapes into built environments, altering the city’s overall land use pattern^1,2,90^. Other land use types, such as forests, vegetation, and bare soil, have decreased by 0.1%, 21.9%, and 14.1%, respectively. The changes in land use classes are shown in Fig. 12.

### Calibration and validation of the hydrological model

Model validation and calibration were carried out on the Ravi River at Balloki. The calibration process includes finding the ideal arrangement of boundaries that best matches the observed and mimicked release. The model was first run in everyday time ventures before being aligned for 1999–2002 and validated for 2003–2005.

**Fig. 13:**
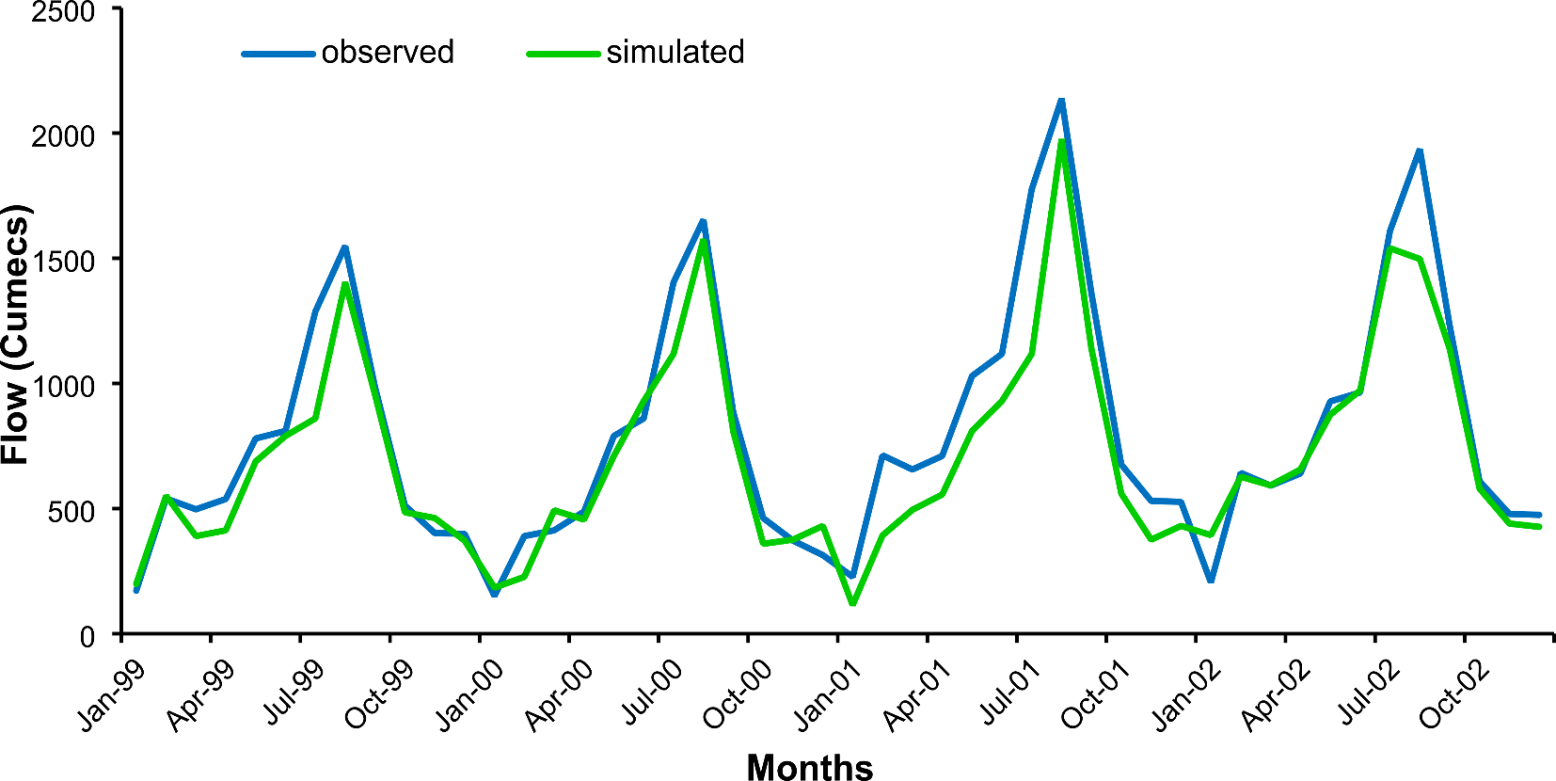
Simulated and Observed Flows for the Calibration Period from 1999–2002.

**Fig. 14:**
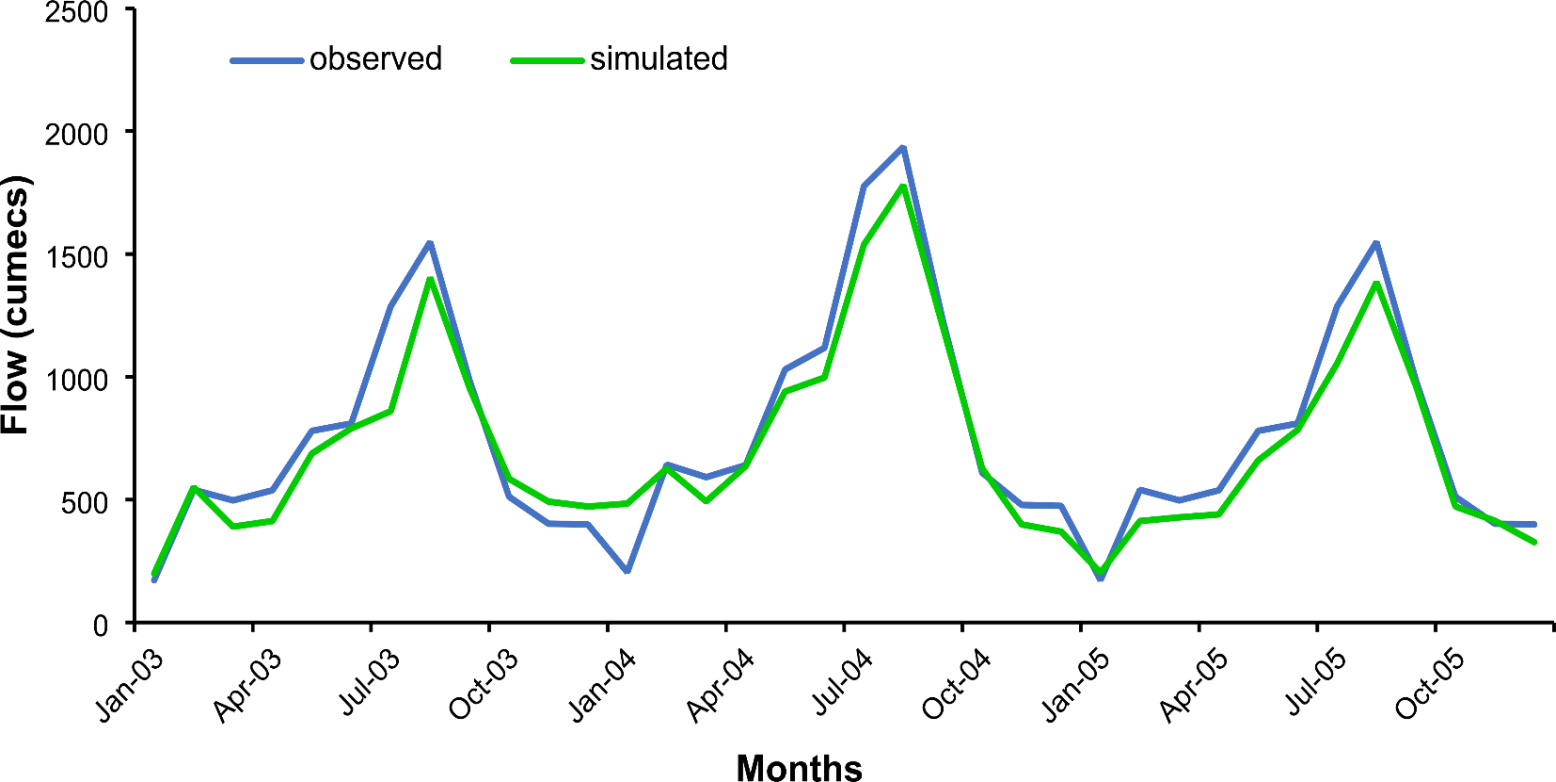
Simulated and Observed Flows for the Validation Period 2003–2005.

The model precisely replicated the day-to-day and month-to-month releases. In Figs. 13 and 14, the validation and calibration results of the SWAT model showed that the observed and simulated discharges were reasonably consistent. Table 4 contains the PBIAS, Nash-Sutcliffe and R^2^ values for calibration and validation.

**Table 4:** Statistics evaluation of the Hydrological Model.

Statistical [arameters	Calibration	Validation
NSE	0.85	0.87
R^2^	0.83	0.81
RMSE	10.01	7.2

### Impact assessment

Two scenarios were considered to assess the impact of climate and land use change on the hydrological response of the study area, namely, scenario I (Hydrological Response under Future Climate and Current Land Use) and scenario II (Hydrological Response under Future Climate and Future Land Use).

**Fig. 15:**
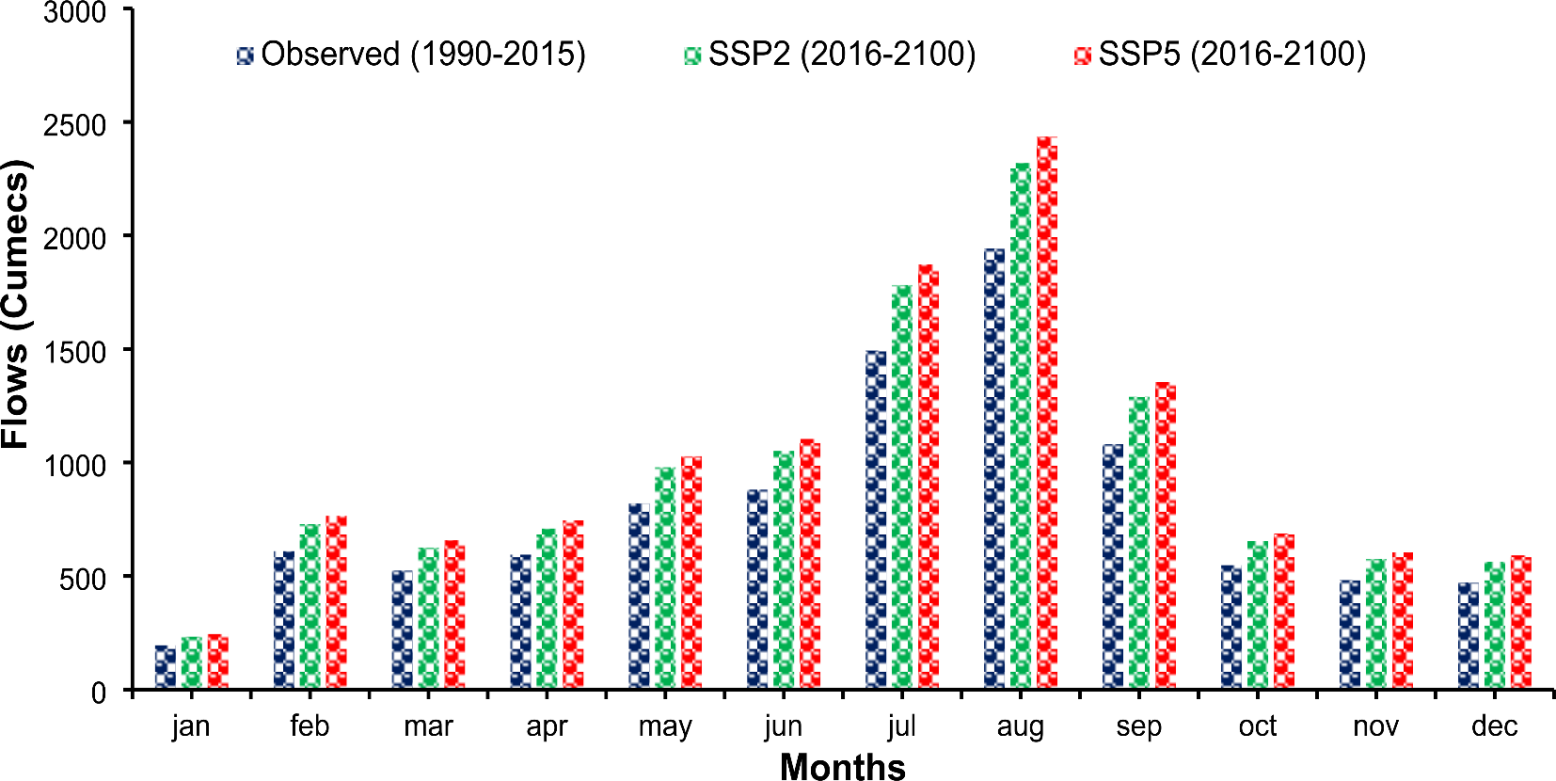
Comparison of baseline and future flows at the Ravi River under Scenario I.

*Scenario I: Hydrological response under future climate and current land use scenarios* The results revealed that stream discharge is expected to increase from 803.25 cumecs in the baseline period (1990–2015) to 959.08 cusecs (an increase of 19.4%) and 1007.28 cumecs (an increase of 25.4%) under SSP2 and SSP5, respectively, in the time frame of 2016–2100. The temporal changes in the mean monthly flows at Balloki are also presented in Fig. 15 under the SSP 2 and SSP 5 scenarios. Both SSPs predict an increase in flow during the whole year.

**Fig. 16:**
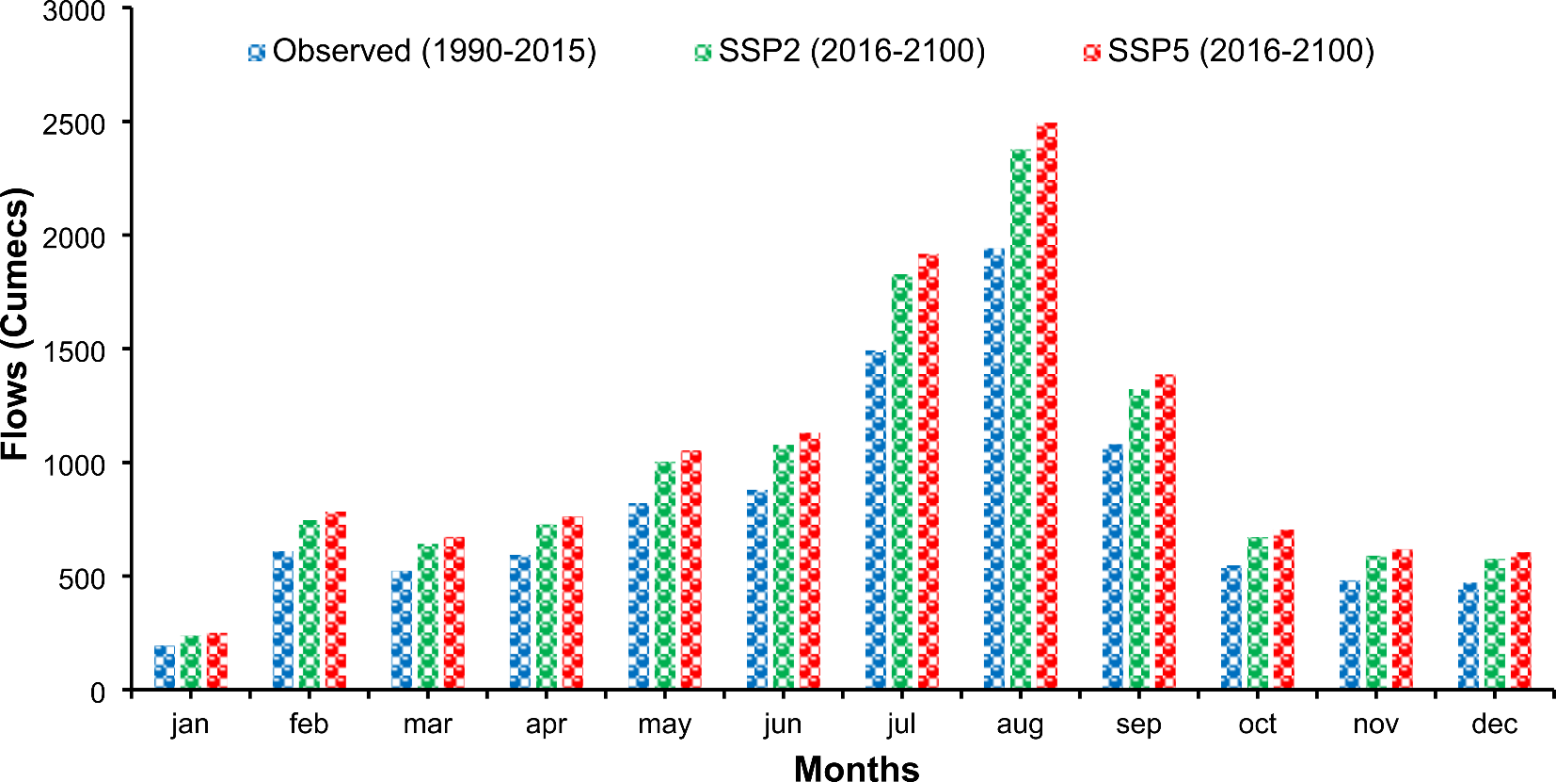
Comparison of baseline and future period flows at the Ravi River under Scenario II.

*Scenario II: Hydrological response under future climate and future land use scenarios* According to the land use patterns, from 2020 to 2100, Lahore’s built-up area and water body area will increase by 31.7% and 4.3%, respectively. Compared with the other land use groups, the amounts of bare soil, forest, and other types of vegetation decreased by 0.1%, 21.9%, and 14.1%, respectively. The stream discharges are supposed to increase from 803.25 cumecs in the baseline period (1999–2015) to 983.18 cumecs (an increment of 22.4%) and 1021.37 cumecs (an increment of 28.4 in the time frame of 2016–2100 under SSP2 and SSP5, respectively) (Fig. 16). When comparing the flow increase rates under the two scenarios, we find that the river flow increase rate is more evident in scenario II. This is attributed to the increase in the built-up area, which prevents water infiltration and increases flow rates. The results also revealed increasing river discharge on a monthly basis throughout the year.

### Contemporary situation of groundwater in Lahore

**Fig. 17:**
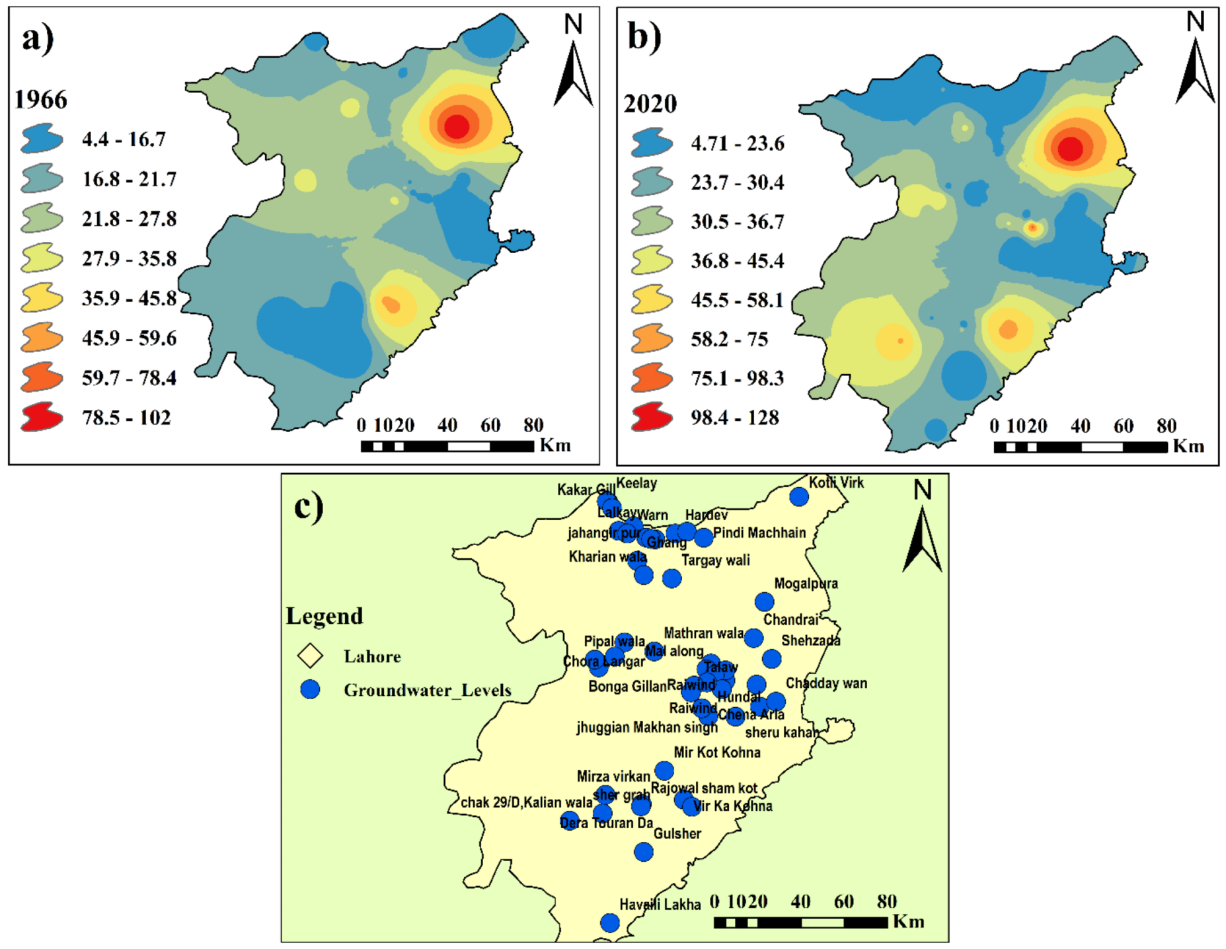
Groundwater depth variation in the Lahore reservoir in 1996 and 2020. Maps created using ArcGIS version 10.8.2 ^53^.

The fluctuations in water depth over the research period were compared and analyzed via maps. Figure 17 shows the groundwater fluctuation maps for the years 1996 and 2020.

### Changes in groundwater depth

**Fig. 18:**
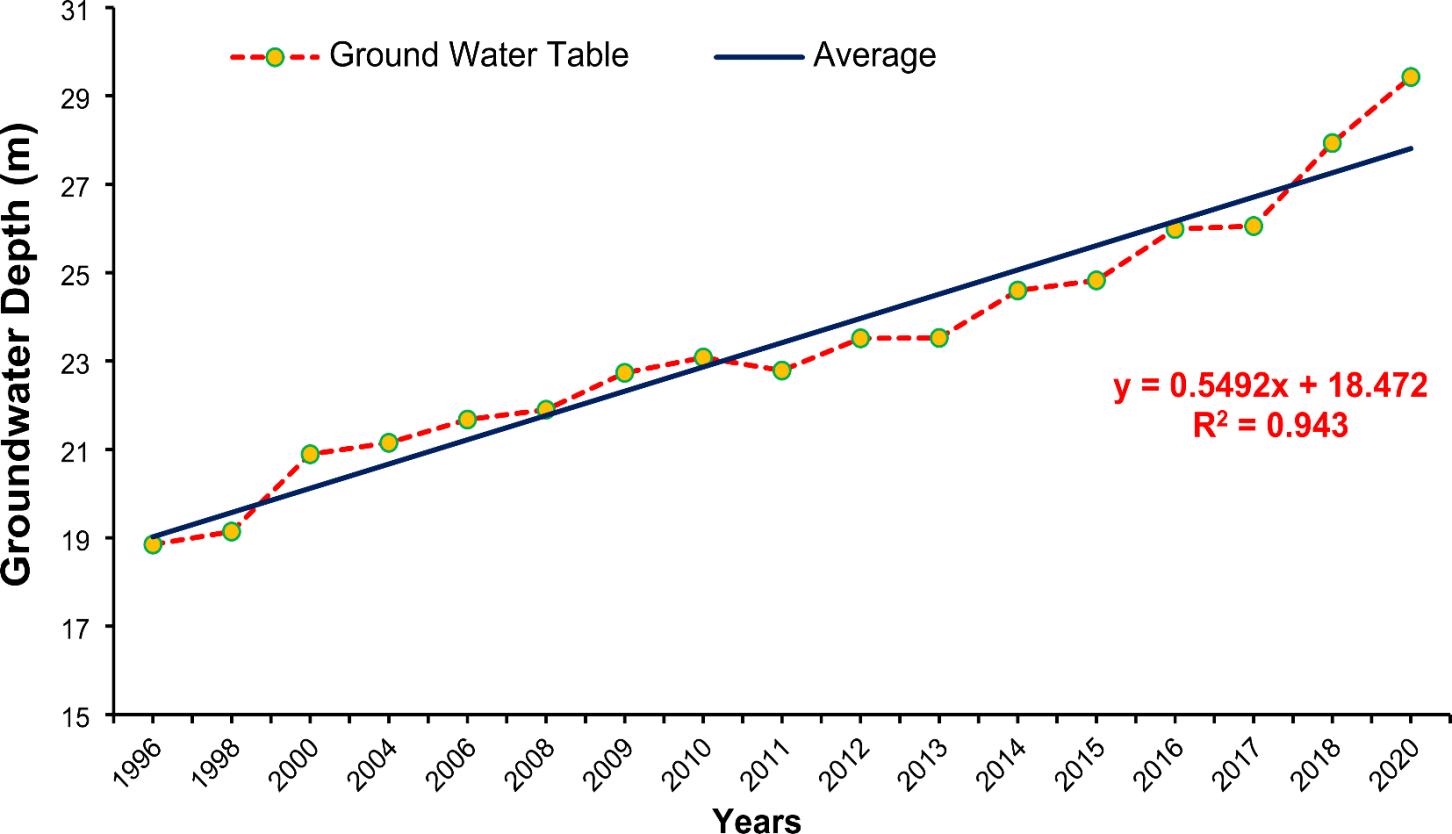
Trend of average depth to groundwater table for Lahore Lake from 1996 to 2020.

Changes in groundwater depth were assessed based on the data collected from different areas of Lahore for 1996 and 2020. The groundwater depth decreases with changes in land use, particularly as built-up areas expand. This expansion leads to increased surface sealing, reducing infiltration and recharge of groundwater. Consequently, as urbanization progresses, the demand for groundwater rises, exacerbating its depletion. Moreover, the heightened demand for water resources within urbanized regions further accelerates this depletion^90,91^. The results are plotted on the map in Table 5. The analysis revealed that the water table has changed throughout the study area. Because of the excessive drawdown of water, a continuous decrease in groundwater levels was observed. Levels fell by 10–14 m from 1996 to 2020, as shown in Fig. 18. The water levels estimated at various locations were averaged to derive a single value for each year spanning from 1996 to 2020.

**Table 5:** Depth of Water table variations (1996–2020).

Locations	Coordinates		Depth of Water Table (m)		
	Latitude	Longitude	Year (1996)	Year (2020)	Change
Bonga Gillan	30°49’7.00″ E	73°55′16.00"N	24.92	34.38	-9.46
Chadday Wan	31°8′4.90″ E	74°9′4.90″ N	11.88	21.96	-10.08
Chora Langar	31°15′1.70″ E	74°6′5.00″ N	19.09	25.50	-6.41
Dera Jarman Wala	31°8′3.90″ E	74°9′5.50″ N	25.72	32.83	-7.11
Hardev	31°10′4.30″ E	74°21′6.00″ N	13.81	16.83	-3.02
Jahangir Pur	31°8′3.30″ E	74°15′49.00"N	15.46	19.58	-4.12
Jhuggian Makhan Singh	31°15′2.90″ E	74°20′22.00"N	14.58	14.17	0.41
Mogalpura	30°56′4.80″ E	74°6′2.20″ N	27.16	39.50	-12.34
Keelay	30°49′1.00″ E	74°6′2.10″ N	13.08	24.67	-11.59
Kharian Wala	30°39′1.20″ E	73°55′53.00"N	34.42	38.13	-3.71
Kotli Virk	30°50′3.20″ E	74°4′3.40″ N	19.42	25.24	-5.82
Lalkay	30°47′3.50″ E	73°46′56.00"N	17.70	24.08	-6.38
Mathran Wala	31°11′4.70″ E	74°24′36.00"N	17.17	17.58	-0.41
Mir Kot Kohna	30°49′3.50″ E	74°55′26.00"N	20.20	25.50	-5.3
Qila Gian Singh	31°15′5.80″ E	74°9′3.50″ N	17.50	6.29	11.21
Raiwind	30°23′4.90″ E	73°48′34.00"N	10.53	25.00	-14.47
Ram Ka Khuh	30°45′5.90″ E	73°39′48.00"N	15.88	24.29	-8.41
Targay Wali	31°13′4.90″ E	74°6′8.30″ N	18.63	23.33	-4.7
Warn	31°10′2.20″ E	74°8′2.80″ N	20.96	21.66	-0.7

**Fig. 19:**
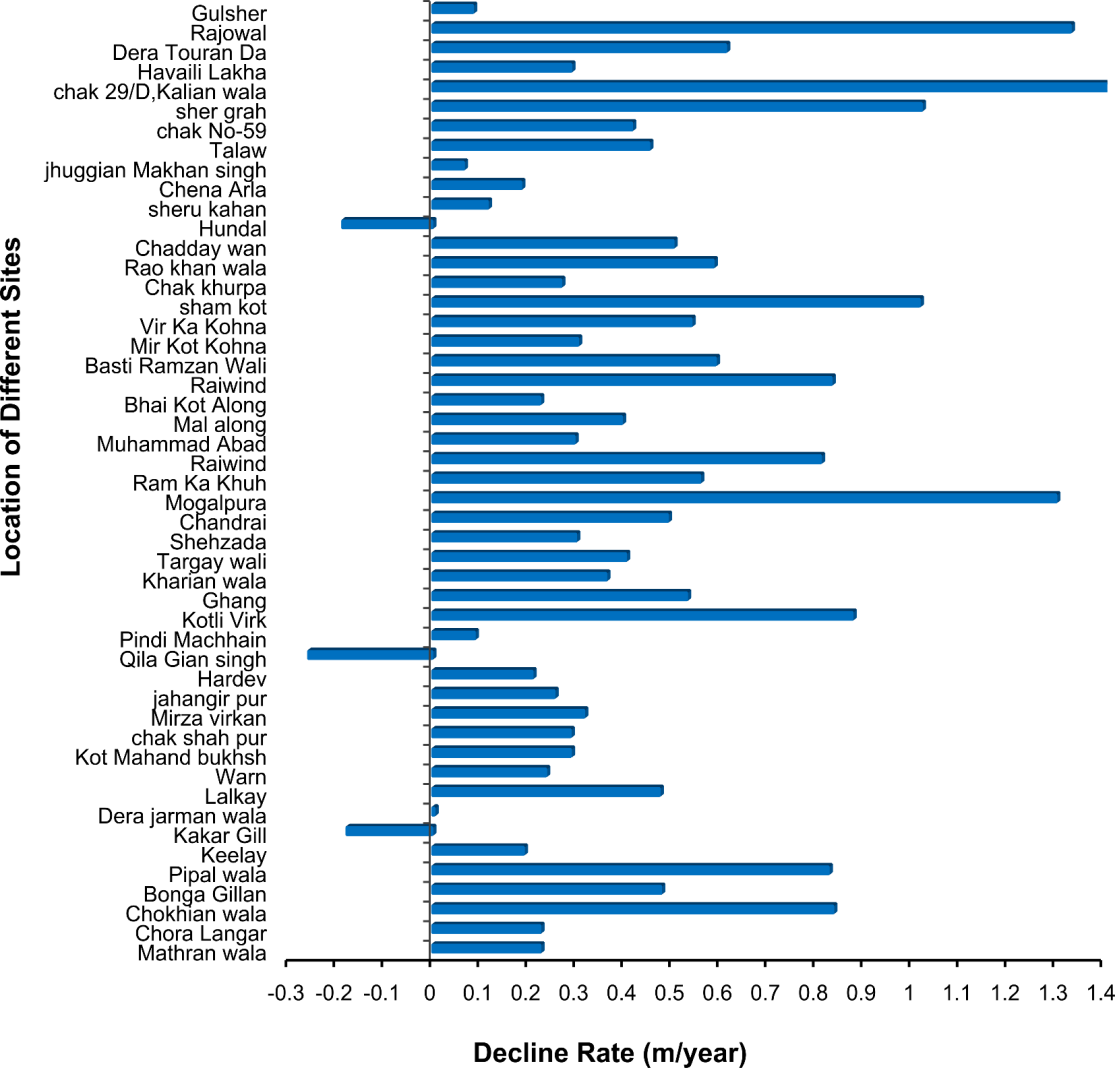
Lahore groundwater depletion rate.

The average estimated groundwater depth in 2020 was 29.43 m. The average groundwater depletion rate was approximately 0.8 m per year, as shown in Fig. 19. The groundwater depletion rate varies from 0.4 m to 0.9 m. An increase of 28.3% in the built-up area over three decades (1995–2020) is enormous. Most of the changes occurred at the expense of vegetation, which decreased by approximately 18.6% from 1995 to 2020. Because of the decrease in vegetated areas, environmental problems can also occur. Additionally, because of the highly built-up areas in Lahore, there is significant variation in land use. The temporal changes in built-up area for the years 1995, 2000, 2010, 2015, 2018, and 2020 are shown in Fig. 9. A comparison with the research of Afzal et al.^92^ revealed that the groundwater level is decreasing by 1.27 m per year.

## Discussion

The Ravi River, one of the five major rivers in the Punjab region, flows through the northern part of Lahore on its western side, serving as a vital water source for the city’s residents, agriculture, and industries^93^. To understand the impact of climate change on river flow patterns, CMIP6 GCM has been utilized, providing crucial insights into potential future scenarios^4^. This study aims to evaluate the effects of projected land cover and climate change on the flow patterns of the Ravi River. This assessment was conducted by using the output from the reliable MPI-ESM1-2-HR GCM model to calibrate a hydrological model. The calibrated model was then employed to analyze the temporal implications of anticipated land cover and climate changes on the basin’s hydrological characteristics. For the base period (1990–2020), the downscaled precipitation and temperature forecasts from the GCM (MPI-ESM1-2-HR) showed a satisfactory alignment with gauge-based readings^6^. This accuracy is likely due to the enhanced capability of CMIP6 models in accurately projecting temperature and precipitation patterns in the Balloki Region, as indicated in previous studies. Groundwater is also depleting due to climate and land use changes, as altered precipitation patterns and increased surface runoff reduce natural recharge rates. Additionally, urbanization and agricultural expansion lead to over-extraction, further exacerbating groundwater depletion^70^.

The study reported a significant decline in groundwater levels in Lahore, with an average depletion rate of 0.8 m/year from 1996 to 2020. Ashraf et al.^7^ studied groundwater recharge in the lower Ravi River basin, finding similar depletion trends due to urbanization and reduced recharge, consistent with our findings for Lahore. Kanwal and Ali^48^ reviewed Lahore’s groundwater depletion, reporting comparable depletion rates and attributing them to similar causes, such as urban expansion and over-extraction. Haider et al.^2^ assessed groundwater recharge in Upper Rechna Doab, Pakistan, identifying comparable impacts of land use change on groundwater levels, which parallels the findings in our study regarding Lahore.

The study utilized CMIP6 GCM (MPI-ESM1-2-HR) for climate projections, predicting increases in temperature and precipitation under SSP2 and SSP5 scenarios. Dubey et al.^44^ applied CMIP6 models in India for crop yield assessments, reporting temperature and precipitation increases similar to those projected in the Ravi River basin, validating the use of CMIP6 for regional climate impact assessments. Wang et al.^59^ used CMIP6 models to project rainfall extremes in the Huai River Basin, China, finding an increase in precipitation under warming scenarios, consistent with the Ravi River projections. Kiran et al.^60^ applied CMIP6 projections in the Chenab River basin, Pakistan, observing similar trends of increased precipitation and temperature under future climate scenarios, aligning with the Ravi basin’s projected outcomes.

The Ravi River demonstrated an ongoing pattern of long-term rising throughout the 21st century on annual and seasonal dimensions, per the analysis of GCM outputs (MPI-ESM1-2-HR)^94^. These results show a general pattern of rising temperatures in South Asia and are in line with earlier research done in nearby areas including the Tibetan Plateau and the Chenab River^95,96^. The higher temperatures recorded in the HKH Mountains are partially explained by the increasing concentrations of greenhouse gases and aerosols in the area. Significant temperature spikes in China have been related to increases in atmospheric aerosol concentrations throughout Asia^97^.

From 2020 to 2100, there is predicted to be a surge in yearly precipitation, and throughout that time, there will also be an uptick in flow in the Yellow River basin’s tributaries^98^. More precipitation is predicted, with the summer and fall months seeing the most significant increases. These results are at odds with studies conducted in the upper Cruz and Kelantan rivers in Malaysia, which found variations of rainfall^69^. Furthermore, there is a surprising decrease in summer rainfall in areas affected by the West, such as Iran and Afghanistan^99,100^. Nonetheless, the research’s observations of monthly rainfall patterns are consistent with those seen in the Punjab and KPK valleys^25,101^. The Jhelum River valley in the Himalayan Alps consistently rises in seasonal and annual rainfall. For Bari Doab, close to the Ravi region, Haider et al.^102^ predicted higher seasonal and yearly precipitation. The primary westerly circulation system of the Hindukush Range could be the cause of this uniformity in findings^43^. An additional reason for the reported similarity in precipitation trends can be found in the increasing concentrations of manmade absorption particles in the air over South Asia^28^.

This research has important applications. First, its conclusions can help local governments and legislators create plans to address possible problems with water scarcity by guiding sustainable practices in managing water resources^66^. Furthermore, especially in light of shifting climatic patterns, the findings can direct the development of adaptive methods to guarantee water availability for industrial, agricultural, and drinking water supplies. Furthermore, local communities can use this information as a helpful resource to make educated decisions about water conservation and usage^103^. Additionally, it advances our understanding of how land use dynamics and climate change combine to affect water resources, providing lessons that other areas facing comparable difficulties might learn from. In summary, this study fills a significant vacuum in the literature. It provides stakeholders with the information they need to negotiate the intricate and dynamic terrain of water resource management for the Ravi River and other regions.

## Theoretical and practical repercussions

First, this study advances our theoretical knowledge of the mechanisms of global warming in river basins. By demonstrating that rising maximum and minimum temperatures are accompanied by growing precipitation, it advances our understanding of regional patterns of climate change. Scientists and academics can better grasp the complexity of climate change in these locations by incorporating this information into climate predictions and hypotheses. Furthermore, the research looks at the complex hydrological events that occur in river valleys with mountains, focusing on the relationship between precipitation, temperature, and river flow^104^. This strengthens the conceptual basis for our comprehension of how climate impacts such regions’ water supplies, making it essential for projecting anticipated shifts and creating workable mitigation and adaptation plans.

The results of this investigation have significant practical ramifications. First of all, the evidence of higher flows emphasizes the significance of aggressive control over water assets, implying the necessity of adaptable strategies to efficiently utilize and oversee more water assets. This could entail maintaining environmentally friendly water distribution procedures, putting flood prevention efforts into place, and enhancing reservoir stewardship. Second, the study helps the area become more resilient to climate change. Comprehending the effects of climate change is essential to creating strong adaptation strategies. The development of climate-resilient buildings, such as enhanced flood protection and reservoirs for water, can be influenced by the findings of this investigation. Such infrastructure is necessary to adapt to shifting temperatures and rainfall regimes. Finally, in light of growing urbanization, this study emphasizes the need for prudent zoning. The impact of cities on the flow of rivers increases with their expansion. Urban centers may expand sustainably and these effects can be lessened by implementing feasible options like green infrastructure and sustainable land use techniques. Lastly, the research’s outcomes have implications for governance across various domains, serving as a basis for evidence-based policies targeted at adaptation and mitigation of climate change. Policymakers can use this information to create land use strategies that minimize adverse effects on water resources and write legislation to lower greenhouse gas emissions^105,106^.

In conclusion, this research contributes to our awareness of the changing nature of environmental change and hydrological events while providing helpful advice for overseeing water resources, developing climate preparedness, designing cities, and governance. These observations are especially helpful for the Ravi River watershed and other places dealing with related issues.

## Conclusions

This investigation aims to evaluate the effects of shifting land use and climate on the Ravi River’s discharges. Two scenarios-one with an emphasis on climate change and one involving land cover and CC modifications- were examined. Two common socioeconomic routes (SSP2 and SSP5) covering the years 2020 to 2100 were used as baseline frameworks for the study. GCM worth of data were evaluated to project climate change, and the model with the best performance was chosen. The analysis of streamflows at Ravi Reach in the Head Balloki under future climate and land cover scenarios that are simulated has led to the subsequent outcomes:

- The current built-up area from land use change (1990–2020) in the study area is 15.8% and is expected to increase by 31.7% in the future (2020–2100).
- Precipitation, maximum temperature, and minimum temperature are expected to increase by 10.9%, 12.2%, and 7.8%, respectively, in the SSP2 scenario and 14.87%, 15.97%, and 9.71%, respectively, in the SSP5 scenario from 2015 to 2100.
- The SWAT model has been successfully applied for the assessment of the impact of future land use and climate change on river flow in Ravi Province, with best-fit NSE, R^2^, and PBIAS values of 0.85, 0.83, and 10.01, respectively, for calibration and 0.87, 0.89, and 7.2, respectively, for validation.
- Considering that current land use will not change in the future and under future climate change, the flows of river Ravi are expected to increase by 19.4% and 25.4%, respectively. Considering future land use and future climate change, the flows of river Ravi are expected to increase by 22.4% and 28.4%, respectively, under SSP2 and SSP5 at the end of the 21st century, indicating an increase in future flows of Ravi.
- Based on the observed data from 1996 to 2020, the average groundwater depth increased by 10.58 ft, and the groundwater depletion rate was 0.8 m/year.

The current study showed that the future flows of the Ravi River at Balloki may increase because of land use and climate change; therefore, future planning should consider increased water resources. The study may further be improved by considering other SSP scenarios. To assist leaders in making plans for numerous options, future research should broaden the scope of scenario evaluation to incorporate a broader range of socioeconomic and legislative instances, including different approaches to land use and mitigating the effects of climate change. Incorporating local neighborhoods and interested parties into the study process can also yield insightful results and guarantee that conclusions are adapted to effectively tackle local issues. Future research can produce more solid and valuable insights for sustainable water resource management in the face of changing climate and land use by using an extensive approach that incorporates enhanced reliability of data, cutting-edge modeling approaches, and thorough scenario analysis. The results of this study are essential for developing strategies for advancing the region, carrying out adaptation plans, and overseeing water projects like irrigation and hydropower sustainably.

## Data Availability

We are thankful to the Punjab Irrigation Department (PID: https:// irrigation.punjab.gov.pk/: access date 28 February 2023) and Pakistan Metrological Department (PMD: https://www.pmd.gov.pk/en/: access date 28 February 2023) for providing the data for flows, and climatic parameters utilized to meet the research objectives effectively. No external output data is generated from this research except that used in supporting the results and analysis in the article.
